# On-Target Anti-TGF-β Therapies Are Not Succeeding in Clinical Cancer Treatments: What Are Remaining Challenges?

**DOI:** 10.3389/fcell.2020.00605

**Published:** 2020-07-08

**Authors:** Adilson Fonseca Teixeira, Peter ten Dijke, Hong-Jian Zhu

**Affiliations:** ^1^Department of Surgery, The Royal Melbourne Hospital, The University of Melbourne, Parkville, VIC, Australia; ^2^Oncode Institute and Department of Cell and Chemical Biology, Leiden University Medical Center, Leiden, Netherlands

**Keywords:** cancer therapy, epithelial to mesenchymal transition, exosome, metastasis, signaling, TGF-β, tumor microenvironment

## Abstract

Metastasis is the leading cause of death for cancer patients. During cancer progression, the initial detachment of cells from the primary tumor and the later colonization of a secondary organ are characterized as limiting steps for metastasis. Epithelial-mesenchymal transition (EMT) and mesenchymal-epithelial transition (MET) are opposite dynamic multistep processes that enable these critical events in metastasis by altering the phenotype of cancer cells and improving their ability to migrate, invade and seed at distant organs. Among the molecular pathways that promote tumorigenesis in late-stage cancers, transforming growth factor-β (TGF-β) is described as an EMT master inducer by controlling different genes and proteins related to cytoskeleton assembly, cell-cell attachment and extracellular matrix remodeling. Still, despite the successful outcomes of different TGF-β pharmacological inhibitors in cell culture (*in vitro*) and animal models (*in vivo*), results in cancer clinical trials are poor or inconsistent at least, highlighting the existence of crucial components in human cancers that have not been properly explored. Here we review most recent findings to provide perspectives bridging the gap between on-target anti-TGF-β therapies *in vitro* and in pre-clinical models and the poor clinical outcomes in treating cancer patients. Specifically, we focus on (i) the dual roles of TGF-β signaling in cancer metastasis; (ii) dynamic signaling; (iii) functional differences of TGF-β free in solution vs. in exosomes; (iv) the regulatory effects of tumor microenvironment (TME) – particularly by cancer-associated fibroblasts – on TGF-β signaling pathway. Clearly identifying and establishing those missing links may provide strategies to revitalize and clinically improve the efficacy of TGF-β targeted therapies.

## Introduction

Affecting human populations in the whole world, cancer is a disease that can virtually compromise all biological human tissues. More than 18 million new cases of cancer were expected for 2018 and more than 9 million patients died in the same year (Bray et al., 2018; Ferlay et al., 2019). Other than different factors distinguishing particular cancer types, metastasis is considered to be the most important cause of death related to this disease and patients affected by metastasis at diagnosis can present a reduced survival rate of 60–90% (Hanahan and Weinberg, 2000; Australian Institute of Health and Welfare [AIHW], 2019).

**FIGURE 1:**
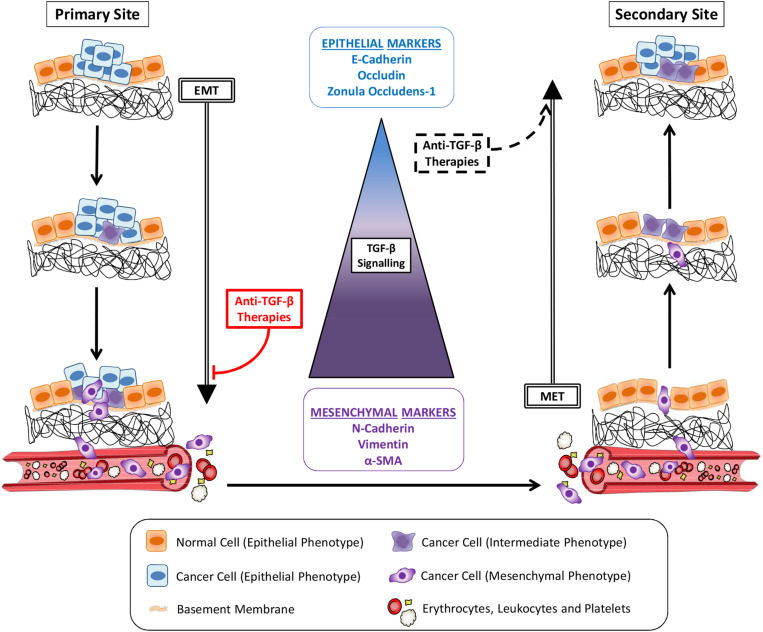
Cancer metastasis and TGF-β signaling. Cancer cells alter their morphology through epithelial-mesenchymal transition (EMT) induced by TGF-β signaling pathway activity, increasing their migratory potential. Invading the basement membrane and the extracellular matrix, tumor cells reach the vasculature (blood or lymph vessels) and become circulating tumor cells (CTCs) after intravasation. Gradually, the magnitudes of TGF-β signaling increase dramatically to enable the EMT-invasion processes. Cancer cells reach a secondary site after extravasation. Following TGF-β signaling reduction and consequent mesenchymal-epithelial transition (MET), cancer cells colonization proceeds to the growth of a metastatic lesion. Anti-TGF-β therapies administered in early stage cancers, before initial invasion, would inhibit metastasis by avoiding EMT. The same strategies used to treat late-stage cancers would also induce MET and seeding of secondary tumors.

Tumor metastasis is a multistep process through which cancer cells leave their primary site to colonize distant organs (Zhou et al., 2014; Ren et al., 2019; Figure 1). In order to migrate and invade, epithelial cancer cells undergo phenotypic alterations to detach from surrounding cells, degrade the basement membrane and remodel the extracellular matrix (ECM) in a process known as epithelial-mesenchymal transition (EMT) (Nieto et al., 2016). These cancer cells will reach blood or lymph vessels and then proceed to vasculature intravasation. Some of the circulating tumor cells (CTCs) which survive into blood or lymph will adhere to vessel walls and escape from the vessel lumen by vasculature extravasation (Kim et al., 2009; Figure 1). Still, while mesenchymal cells present enhanced ability to invade different tissues and proceed to vasculature intravasation/extravasation during metastasis, this phenotype impairs their establishment in a secondary site, limiting the growth of macrometastasis (Figure 1). Thus, after reaching a new organ, neoplastic cells reverse their phenotype through the mesenchymal-epithelial transition (MET), improving their interaction with the microenvironment and increasing their proliferation rate and chance of survival (Chaffer et al., 2006; Biswas et al., 2014). Therefore, the two opposite processes of EMT and MET in metastasis early and late-stages, respectively, are considered to be critical steps in cancer metastasis.

Different molecular pathways are associated with the phenotypic changes observed in metastatic cells, including those mediated by transforming growth factor-beta (TGF-β), epidermal growth factor (EGF), hepatocyte growth factor (HFG), platelet derived growth factor (PDGF), notch, and wnt (Nieto et al., 2016). Among these important pathways, TGF-β signaling is considered to act as a master inducer of EMT, invasion and metastasis by controlling different genes and proteins related to cytoskeleton assembly (Gladilin et al., 2019), cell-cell attachment (Kim et al., 2019) and ECM remodeling (Mori et al., 2015). TGF-β is a secreted dimeric polypeptide that elicits cellular effects via cell surface TGF-β type I and type II receptors (TβRI and TβRII). They have intrinsic serine/threonine kinase activity and activate intracellular (non)SMAD signaling pathways (Hao et al., 2019). Each step in the TGF-β signaling pathway is tightly regulated, and subject to crosstalk with other signaling pathways (Liu et al., 2018). TGF-β signaling pathway is well-characterized and many strategies have been used to interfere with its activity (Colak and Ten Dijke, 2017). Nevertheless, even if the selective inhibition of TGF-β bioavailability, TGF-β/TGF-β receptor interaction or TGF-β receptor kinase activity is efficacious *in vitro* and *in vivo*, outcomes observed for anti-TGF-β therapies in clinical settings are often unsatisfactory. In the next sections, we provide a brief overview of TGF-β signaling pathways (section “TGF-β as a Critical Driver in Cancer Progression”); describe and compare different TGF-β signaling inhibitors used *in vitro*, *in vivo*, and in human patients (section “Anti-TGF-β Therapies and Their Poor Outcomes in Cancer Clinical Trials”); and discuss critical issues in preclinical experiments that so far have been largely ignored/overlooked that could explain the poor outcomes observed in cancer clinical trials (sections “Controlling Metastasis Critical Steps: The Dual Role of TGF-β,” “TGF-β Dynamic Signaling,” “Tumor Microenvironment Regulates TGF-β Signaling,” and “Exosomes as a Mechanism of TGF-β Secretion and Signaling Amplification”). When used in particular studies, TGF-β isoforms are indicated during this discussion, otherwise they are referred as TGF-β if this specificity is not relevant.

## TGF-β as a Critical Driver in Cancer Progression

Until early 1980s, thanks to studies exploring the role of infectious agents on cancer development, the acquisition of a malignant phenotype was greatly associated with a virus-induced reprogramming of normal cells (Stehelin et al., 1976; Levinson et al., 1978). Products of avian, murine and feline tumor viruses’ genomes were shown to drive the malignant transformation of normal cells by the hyperactivation of signaling pathways (Todaro et al., 1976; Levinson et al., 1978; Hackett et al., 1981). In this scenario, the elevated secretion of growth factors was described as an important mechanism able to cause normal fibroblasts transformation, as observed by an increased anchorage-independent growth potential *in vitro* that was intimately associated with cancer cells behavior *in vivo* (de Larco and Todaro, 1978). These molecules later named as transforming growth factors (TGF) were later purified and assigned as TGF-α and TGF-β, being the later characterized as a critical component in the process of malignant transformation (Roberts et al., 1980, 1981; Anzano et al., 1982). Since then, many other related molecules were studied and nowadays TGF-β is part of a protein family of growth factors and cytokines.

Based on similarity in sequence and function, TGF-β family is divided in two subgroups: TGF-βs, activins, and nodals forming one group and bone morphogenetic proteins (BMP)s and anti-muellerian hormone the other. The cellular responses to TGF-β and BMP are highly context-dependent, and have been attributed both anti- and pro-tumorigenic roles in different cancer types and/or stages of cancer progression (Biswas et al., 2008; Zhong et al., 2010; Luwor et al., 2015; Sachdeva et al., 2019; Vollaire et al., 2019). The biphasic role of TGF-β family pathways in cancer were already reviewed in details by others (Lebrun, 2012; Seoane and Gomis, 2017). Among all TGF-β family members, the targeting of TGF-β pathway has been explored most for therapeutic gain in the treatment of cancer patients (Colak and Ten Dijke, 2017; Hao et al., 2019). In this review, therefore, we focus on the TGF-β signaling pathway and selective intervention strategies as a background to discuss problems related to pharmacological inhibitors for TGF-β family members used in preclinical and clinical cancer studies.

### TGF-β Secretion and Activation

The expression TGF-β isoforms (TGF-β1-3) is coordinated in tissues according to physiopathological conditions (Stenvers et al., 2003; Cooley et al., 2014; Denney et al., 2015; Hachim et al., 2018). Importantly, TGF-β is secreted in an inactive form in which the N-terminal sequence (also termed latency-associated peptide, LAP), and a C-terminal sequence (active cytokine) are non-covalently linked (Walton et al., 2010). Dimers of TGF-β:LAP associate with the latent TGF-β binding protein (LTBP) to form the large latent complex (LLC) (Taipale et al., 1994; Walton et al., 2010). While LAP prevents TGF-β activation, LTBP promotes secretion and can mediate the TGF-β association with proteins in ECM. Besides enzymatic cleavage, a non-enzymatic mechanism of TGF-β activation is also reported and relies on the interaction of LLC with integrins. In cells with enhanced contractility, the tension created by cytoskeleton exerts physical forces that unfold LAP and release active TGF-β (Taipale et al., 1994; Shi et al., 2011).

### TGF-β Receptor Signaling Pathways

After secretion and activation, TGF-β ligands bind to heteromeric complexes of type I and type II serine/threonine kinase receptors (i.e., TβRI and TβRII). TβRII is a constitutive active kinase that phosphorylates TβRI upon ligand binding, thereby enabling the transduction of extracellular signal into the cell (Zhu and Sizeland, 1999). The activated TβRI initiates intracellular signaling by phosphorylation of downstream effector molecules. Besides TβRI and TβRII, TGF-β can interact with more abundant auxiliary receptors, e.g., TGF-β type III receptor (TβRIII), that lack an enzymatic intracellular motif (Andres et al., 1992; Stenvers et al., 2003). These co-receptors can enable presentation of TGF-β to TβRI and TβRII and thereby regulate cellular responsiveness (López-Casillas et al., 1993; Stenvers et al., 2003). Moreover, as TGF-β isoforms bind with different affinity to co-receptors, they contribute to isoform specific responsiveness to different cell types (Andres et al., 1992; Itoh et al., 2003).

**FIGURE 2:**
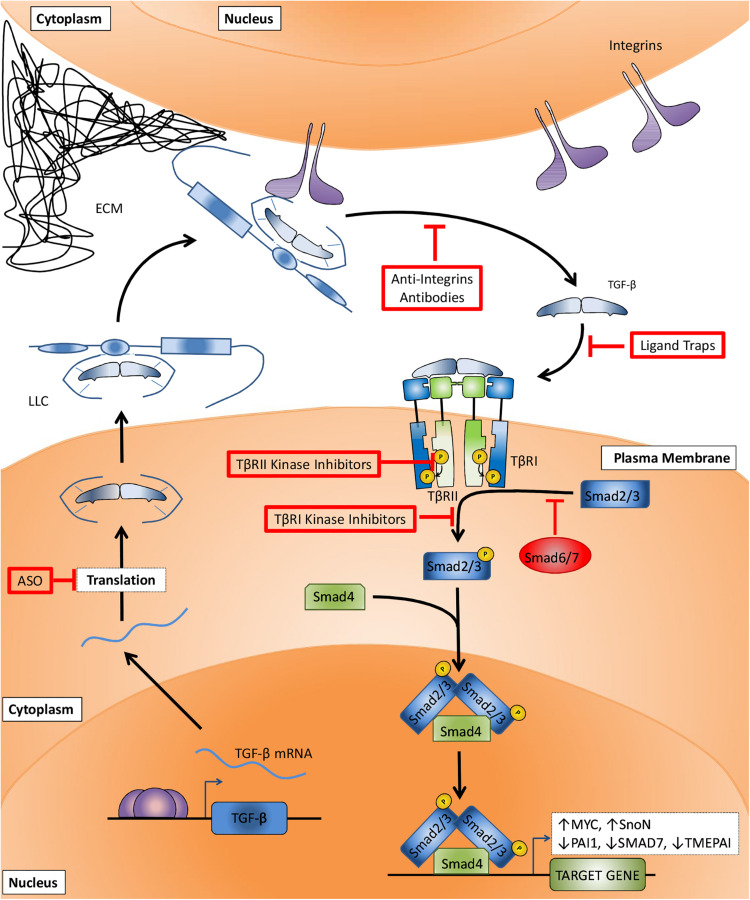
Canonical TGF-β signaling pathway and TGF-β signaling targeting therapies. After TGF-β mRNA translation (step I) and secretion, the large latent complex (LLC) composed of TGF-β, latency associated peptide (LAP), and latent TGF-β binding protein (LTBP) is deposited to the extracellular matrix (ECM). The interaction between LTBP and integrins increases TGF-β:LAP dissociation and TGF-β activation (step II). TGF-β binding to surface receptors (step III) is followed by TβRII-mediated TβRI transphosphorylation (step IV). The signaling is then transduced to cytosol by TβRI-induced phosphorylation of SMAD2 and 3 (step V), followed by their association with SMAD4, accumulation in the nucleus and regulation of target genes transcription. Anti-TGF-β therapies target critical steps in order to impair TGF-β signaling. Antisense oligonucleotides (ASOs) prevent the translation of TGF-β mRNA (step I). Anti-integrins prevent TGF-β activation (step II). Ligand traps avoid cytokine binding to its receptors (step III). TβRII and TβRI kinase inhibitors block type II-mediated type I receptor phosphorylation (step IV) and type I-mediated SMAD2 and 3 phosphorylation (step V), respectively.

SMADs act as specific effectors downstream of activated TGF-β family type receptors. In the canonical TGF-β-SMAD signaling pathway (Figure 2), TβRI kinase induces the phosphorylation of a Sma- and Mad- related (SMAD) 2 and 3. BMP type I receptors mediate the phosphorylation of distinct set of R-SMADs, i.e., SMAD1, 5, and 8. Common SMAD (Co-SMAD), i.e., SMAD4 binds to phosphorylated R-SMADs to form heteromeric complexes that accumulate in the nucleus and control target gene expression. Another set of SMADs are the inhibitory SMADS (I-SMADs), i.e., SMAD6 and 7. I-SMADs antagonize signal transducing SMADs via multiple mechanisms, including direct competition with R-SMADs for SMAD4, and recruitment of ubiquitin ligases that drive type I receptor polyubiquitination and degradation. Besides canonical SMAD signaling, TGF-β family type I receptors can also initiate so-called non-SMAD signaling pathways that follow intracellular downstream routes, controlling the stability, activity and expression of genes and proteins (Nakao et al., 1997; Shi et al., 1997; Itoh et al., 2003; Zhang et al., 2007; Fleming et al., 2013). Different studies have demonstrated for example the TβRI-induced activation of mitogen-activated protein kinase (MAPK) (Tang et al., 2019), and phosphatidylinositol-4,5-bisphosphate 3-kinase (PI3K)/protein kinase B (PKB/AKT) pathways (Kattla et al., 2008).

By activating its canonical and non-canonical pathways, TGF-β controls multiple processes in cell homeostasis. In non-malignant cells and in early stage cancers, TGF-β exerts a tumor-suppressive role inducing cell cycle arrest and apoptosis. In fact, inactivating mutations in TGF-β receptors and SMADs are frequently observed in cancers (e.g., colorectal, pancreas, and lung cancers) (Hahn et al., 1996; de Jonge et al., 1997; Shi et al., 1997; Zhang et al., 2003; Biswas et al., 2008; Fleming et al., 2013). Nonetheless, many other cancer types, such as brain, breast and skin, bypassing TGF-β cytostatic or pro-apoptotic effects through mutations in different pathways (e.g., PI3K/AKT), become invasive by subverting TGF-β activity to their own benefit (Biswas et al., 2014; Yang et al., 2016). In this scenario, TGF-β tumor-promoter role contributes directly and indirectly with metastatic potential of cancer cells. Directly, TGF-β induces EMT to support migration and invasion of cancer cells as previously mentioned. Indirectly, TGF-β acts on distinct elements of tumor microenvironment, suppressing immune surveillance, promoting angiogenesis and activating cancer-associated fibroblasts that will further contribute to metastasis (Itoh et al., 2009; Liu et al., 2016a, b; Stockis et al., 2017).

The accumulated evidences about critical steps in TGF-β signaling activation combined to the relevance of TGF-β in cancer progression led to the development of multiple strategies to abrogate its activity. Anti-TGF-β therapies have been extensively investigated, but despite their very well-established efficacies and ability to act on target, clinical trials are still unable to reproduce these outstanding results obtained *in vitro* and *in vivo* (Ahmadi et al., 2019). On the next section we present different mechanisms to block the TGF-β signaling pathway and a brief compilation of preclinical and clinical data obtained from studies using TGF-β inhibitors in order to contextualize the missing points when these therapies are translated from bench-to-bedside.

## Anti-TGF-β Therapies and Their Poor Outcomes in Cancer Clinical Trials

Multiple strategies have been developed to target the TGF-β signaling pathway, including interference with activation of latent TGF-β, ligand-receptor interactions, and receptor kinase inhibitors. While *in vitro* and preclinical models have been clearly successful, so far the outcomes from clinical trials to treat different types of cancers have frequently shown (at best) only a minor survival benefit and even sometimes adverse effects. One reason for poor clinical translation may well be that the preclinical data may suffer from publication bias for positive results, and that the animal models used in these studies poorly reflect the cancers developed in patient. In addition, with TGF-β being a multifunctional cytokine of key importance to the maintenance of tissue homeostasis, targeting of TGF-β signaling has been associated with on-target cardiovascular toxic side effects and formation of benign tumors (Colak and Ten Dijke, 2017). Inappropriate patient selection in clinical trials may also contribute to the inability to demonstrate favorable survival benefit. Moreover, as targeting TGF-β will not kill the cancer cell, but is aimed at inhibiting invasion and metastasis, it will have to be used with other agents that do kill cancer cells (Bhola et al., 2013; Zhu et al., 2018). Furthermore, targeting TGF-β signaling has been frequently aimed at inhibiting cancer cell invasion and metastasis, but inhibition of immune evasion by blocking the potent immune suppressive function of TGF-β might actually be more important for anti-cancer activity of TGF-β targeting agents (Ghiringhelli et al., 2005; Yang et al., 2008; Rong et al., 2016; Xia et al., 2017; Biswas et al., 2019). Thus, drugs used so far do not recapitulate preclinical data and the outcomes reported for these tests are inconsistent among patients as discussed in the following sections. In order to understand the mechanisms of action on which these strategies are based and the possible reasons for their failure in clinical tests, four categories of anti-TGF-β therapies will be further discussed: (i) antisense oligonucleotides (ASOs), (ii) anti-integrins, (iii) ligand traps, and (iv) kinase inhibitors (Figure 2).

### Antisense Oligonucleotides

ASOs are designed to bind to and prevent TGF-β mRNA translation, consequently decreasing its expression. Tests in mesothelioma and prostate cancer cells lines, for example, demonstrated its effectiveness in dramatically reducing TGF-β protein expression and inhibiting anchorage-independent growth (Fitzpatrick et al., 1994; Matthews et al., 2000). Further experiments *in vivo* showed reduced tumor growth in animals subjected to ASOs treatments and these results were associated with impairment of TGF-β-mediated immune suppression (Fitzpatrick et al., 1994; Matthews et al., 2000).

Based on its proven specificity observed in preclinical models, ASOs have progressed to clinical trials. AP 12009 (or Trabedersen), an ASO targeting TGF-β2 mRNA, was used to treat multiple cancer types. The safety of Trabedersen was demonstrated in phase I trials in patients with pancreas, colon, and skin cancers (NCT00844064). In a phase II trial, Trabedersen was administered to patients with glioblastoma and anaplastic astrocytoma (NCT00431561), achieving a particularly interesting outcome: compared to patients treated with standard chemotherapy (i.e., Temozolomide or Procarbazine/Lomustine/Vincristine), patients submitted to this ASO appeared to exhibit an improvement in cognitive functions. Nevertheless, the same study failed to demonstrate increased antitumor responses in patients treated with Trabedersen compared to patients treated with standard chemotherapy. Finally, the only phase III clinical trial using Trabedersen, also to treat brain cancer patients (NCT00761280), has been terminated by its inability to recruit the projected number of patients and only descriptive analyses are available. Table 1 summarizes main results obtained in cancer clinical trials using ASOs.

**TABLE 1:** Overview of anti-TGF-β therapies based on antisense oligonucleotides used in cancer clinical trials.

Drug (Target)	Clinical trial (Phase)	Status	Cancer type	Patients enrolled	Arms	Outcomes
AP 12009 (TGF-β2)	NCT00431561 (Phase II)	Completed	Glioblastoma and anaplastic astrocytoma	141	AP 12009 (10 μM) AP 12009 (80 μM) Temozolomide or procarbazine, lomustine, and vincristine	Improved PFS Improved OS (Results for responders regardless drug concentration administered)
AP 12009 (TGF-β2)	NCT00761280 (Phase III)	Terminated	Glioblastoma and anaplastic astrocytoma	27	AP 12009 (10 μM) Temozolomide or carmustine or lomustine	NA
AP 12009 (TGF-β2)	NCT00844064 (Phase I)	Completed	Melanoma, pancreatic and colorectal neoplasms	62	Single-arm: AP 12009 (dose escalation)	NA

*PFS, progression-free survival; OS, overall survival. NA, not available.*

### Anti-integrins

TGF-β activation by dissociation from LAP is a crucial step that precedes its binding to TβRI/II. As mentioned previously, different mechanisms work toward TGF-β activation, binding of LTBP to integrins is considered one of them to greatly improve the activation process. In fact, integrins expression is associated with elevated availability of activated TGF-β and consequent increase of EMT, migration and invasion *in vitro* for many cancer cell lines (Roth et al., 2013; Moore et al., 2014; Dutta et al., 2015; Takasaka et al., 2018). Furthermore, the activation of TGF-β signaling pathway is shown to induce integrins expression leading to a positive feedback (Mori et al., 2015; Liu and Shang, 2020; van Caam et al., 2020). Consequently, many strategies targeting TGF-β signaling by blocking integrin-mediated TGF-β activation were developed and tested in preclinical models. For instance, antibodies blocking integrins (e.g., 10D5 and 264RAD) efficiently impair the growth of primary and secondary tumors in models of breast and prostate cancers, though the effects exerted by these therapies could also be related to reduced TGF-β-mediated immunosuppression and angiogenesis (Moore et al., 2014; Dutta et al., 2015).

Seven cancer clinical trials exploring the effects of integrins inhibitors were conducted so far, but two were terminated (NCT01122888 and NCT02337309) before prematurely conclusion and three others do not present results publicly available (NCT00721669, NCT00284817, NCT00635193). The two remaining studies evaluated the use of EMD 121974 (or Cilengitide), an antibody targeting integrins αvβ3 and αvβ5, to treat patients with head and neck squamous cell carcinoma (phases I and II, NCT00705016) and glioblastoma (phase III, NCT00689221). Unfortunately, both trials report that administration of Cilengitide did not result in improved antitumor activity or increased overall survival compared with standard chemotherapies. Table 2 shows an overview of clinical studies that evaluated integrin inhibitors to treat cancer patients.

**TABLE 2:** Overview of anti-TGF-β therapies based on integrin inhibitors used in cancer clinical trials.

Drug (Target)	Clinical trial (Phase)	Status	Cancer type	Patients enrolled	Arms	Outcomes
EMD 121974 (Integrins αvβ3 and αvβ5)	NCT01122888 (Phase I)	Terminated	Adult giant cell glioblastoma, adult glioblastoma, adult gliosarcoma, adult solid neoplasms and recurrent adult brain neoplasms	41	Sunitinib + EMD 121974 Sunitinib	NA
EMD 121974 (Integrins αvβ3 and αvβ5)	NCT00705016 (Phases I/II)	Completed	Head and NeckSquamous Cell Carcinoma	184	Cilengitide (2000 mg) once weekly + cetuximab + 5-FU + cisplatin Cilengitide (2000 mg) twice weekly + cetuximab + 5-FU + cisplatin Cetuximab + 5-FU + Cisplatin	No improvement in PFS No improvement in OS
EMD 121974 (Integrins αvβ3 and αvβ5)	NCT00689221 (Phase III)	Completed	Glioblastoma	545	Cilengitide + temozolomide + radiotherapy Temozolomide + radiotherapy	No improvement in PFS No improvement in OS
SF1126 (Integrin-targeted PI3 kinase)	NCT02337309 (Phase I)	Terminated	Neuroblastoma	4	Single-arm: SF1126	NA
IMGN388 (Integrins αv)	NCT00721669 (Phase I)	Completed	Melanoma, breast carcinomas, lung carcinomas and ovary carcinomas	60	Single-arm: IMGN388	NA
MEDI-522 (Integrin αvβ3)	NCT00284817 (Phases I/II)	Completed	Colorectal cancer	17	MEDI-522 (D0: 4 mg/kg; W1–W51: 1 mg/kg) MEDI-522 (D0: 4 mg/kg; W1–W51: 2 mg/kg) MEDI-522 (D0: 6 mg/kg; W1–W51: 2 mg/kg) MEDI-522 (D0: 6 mg/kg; W1–W51: 3 mg/kg)	NA
M200 (Integrin α5β1)	NCT00635193 (Phases I/II)	Completed	Ovarian cancer and primary peritoneal cancer	138	Liposomal doxorubicin (40 mg/m^2^) + M200 (7.5 mg/kg) Liposomal doxorubicin (40 mg/m^2^) + M200 (15.0 mg/kg) Liposomal doxorubicin (40 mg/m^2^)	NA

*5-FU, 5-fluoracil; D0, First day of treatment; W1–W51, weeks 1–51 of treatment; PFS, progression-free survival; OS, overall survival; NA, not available.*

### Interfering With Ligand-Receptor Interactions

TGF-β signals when the active cytokine binds to surface receptors that will further transduce the signal to cytoplasm and two main strategies were developed so far as ligand trap to prevent this step: (i) administration of antibodies against ligand or its receptors, and (ii) the use of soluble TGF-β receptors (sRII or sRIII) or receptors fused to immunoglobulins (TβRII:Fc) as ligand sequesters. Many molecules designed as ligand traps have been characterized *in vitro* and *in vivo.* Their ability to reduce the availability of the active cytokine, diminish SMAD2/3 phosphorylation and decrease the expression of TGF-β target genes, support their on-target activity (Ganapathy et al., 2010). For instance, treatments with 1D11 or 2G7 (monoclonal anti-TGF-β antibodies) were shown to reduce the metastatic burden and angiogenesis in breast cancer models and further experiments associated these results to an increased cytotoxicity exhibited by natural killer (NK) cells (Arteaga et al., 1993; Ganapathy et al., 2010; Biswas et al., 2011). Similar results were obtained by employing antibodies raised against the extracellular domain of TGF-β receptors (particularly against TβRII), reducing the growth of primary and secondary tumors as well as increasing the numbers of NK and cytotoxic T cells (Zhong et al., 2010). Also, mice models treated with the sRIII (Bandyopadhyay et al., 1999) or TβRII:Fc (Muraoka et al., 2002; Yang et al., 2002) showed a reduced number of metastases in different organs analyzed (i.e., lung, liver, and pancreas). This approach has recently been expanded by fusing the extracellular domain of TβRII with an anti-programmed cell death ligand-1 (PD-L1) antibody to obtain a bifunctional therapy and circumvent the immunosuppression commonly observed in solid tumors. *In vitro*, this bifunctional therapy (M7824) was demonstrated to increase the lysis of urothelial carcinoma cells by T cells compared to effects of anti-PD-L1, a result that was associated to the upregulation of molecules involved in immunogenic modulation (i.e., intercellular adhesion molecule 1/ICAM-1, carcinoembryonic antigen/CEA, and Fas cell surface death receptor/FAS) (Grenga et al., 2018). A similar pattern has also been demonstrated for this strategy *in vivo*, in which the administration of an anti-PD-L1-TβRII reduced tumor burden and promoted activation of CD8^+^ T lymphocytes and NK cells in breast and colorectal cancer models (Ravi et al., 2018).

Multiple observations in preclinical models led ligand traps to cancer clinical trials, but different from animal models, results in humans have been inconsistent. The TGF-β sequester GC1008 (also known as Fresolimumab), one of the best characterized monoclonal anti-TGF-β1-3 antibodies was used in patients with renal cell carcinoma (phase I, NCT00923169), melanoma (phases I and II, NCT00923169), glioma (phase II, NCT01472731), mesothelioma (phase II, NCT01112293), and breast cancer (phase II, NCT01401062). Even though a relationship between safety and antitumor activity was shown, it was also observed a decreased expression of activating surface proteins in NK cells (i.e., CD226 and CD244) which could impair therapy effects and partially explain why most patients treated in these studies did not present improved overall survival. Currently two other clinical trials using anti-TGF-β antibodies are recruiting: a phase I/Ib trial (NCT02947165) using NIS793 (anti-TGF-β) to evaluate its safety and tolerability as a single agent or in combination with PDR001 (anti-programmed cell death-1, or PD-1), and another phase I study (NCT03192345) using SAR439459 (anti-TGF-β) to evaluate safety, pharmacokinetics, pharmacodynamics and antitumor activity as a monotherapy or in combination with Cemiplimab (anti-PD-1) in multiple cancers. Similar to antibodies targeting the ligand, many problems were also observed when using anti-TGF-β receptors antibodies. For example, a study evaluating the safety of LY3022859 (anti-TβRII) to treat solid tumors (NCT01646203) failed in establishing its maximum tolerated dose, restricting its usage in other phases. Finally, the only clinical trial (phase I) proposed so far to treat cancer patients by blocking TGF-β signaling pathway by the use of soluble TGF-β receptors is still recruiting patients. This study (NCT03834662) will evaluate AVID200 safety, tolerability, and dose-limiting toxicities in advanced or metastatic solid cancers. Results obtained in clinical trials evaluating the interference between ligand-receptor interactions in the treatment of cancer patients are summarized in Table 3.

**TABLE 3:** Overview of anti-TGF-β therapies based on the interference between ligand-receptor interactions used in cancer clinical trials.

Drug (target)	Clinical trial (phase)	Status	Cancer type	Patients enrolled	Arms	Outcomes
GC1008 (TGF-β1 and TGF-β2)	NCT00923169 (Phase I)	Completed	Renal cell carcinoma and melanoma	22	GC1008 (10 mg/kg) GC1008 (15 mg/kg)	Highest safe dose: 15 mg/kg
GC1008 (TGF-β1 and TGF-β2)	NCT01472731 (Phase II)	Completed	Glioma	12	Bioimaging with 89Zr-GC1008 (37 MBq total) Treatment with GC1008 (5 mg/kg)	NA
GC1008 (TGF-β1 and TGF-β2)	NCT01112293 (Phase II)	Completed	Mesothelioma	14	Single-arm:GC1008 (3 cycles)	NA
GC1008 (TGF-β1 and TGF-β2)	NCT01401062 (Phase II)	Completed	Metastatic breast cancer	23	GC1008 (1 mg/kg) + radiotherapy GC1008 (10 mg/kg) + radiotherapy	No improvement in abscopal effect Improved OS in arm II
NIS793 (TGF-β)	NCT02947165 (Phase I)	Recruiting	Breast, lung, hepatocellular, colorectal, pancreatic and renal cancers	220	NIS793 NIS793 + PDR001	NA
SAR439459 (TGF-β1, TGF-β2 and TGF-β3)	NCT03192345 (Phase I)	Recruiting	Advanced solid tumors	225	SAR439459 (dose escalation) SAR439459 (dose expansion) SAR439459 (dose escalation) + cemiplimab SAR439459 (dose expansion) + cemiplimab	NA
LY3022859 (TβRII)	NCT01646203 (Phase I)	Completed	Advanced solid tumors	14	IMC-TR1 (1.25 mg/kg) IMC-TR1 (dose escalation – 12.5 to 1600 mg) IMC-TR1 (dose escalation – 800 to 1600 mg)	DLT reported TEAE reported SAE reported
AVID200 (TGF-β1 and TGF-β3)	NCT03834662 (Phase I)	Recruiting	Malignant solid tumors	36	AVID200 (180 mg/m^2^) AVID200 (550 mg/m^2^) AVID200 (1100 mg/m^2^)	NA

*OS, overall survival; DLT, dose-limiting toxicity; TEAE, treatment emergent adverse events; SAE, serious adverse event; NA, not available.*

### Kinase Inhibitors

Kinase inhibitors block the binding of ATP to TGF-β receptors, reducing their kinase activity and limiting downstream signaling transduction. Similar to ligand traps, their ability to specifically target and impair TGF-β signaling pathway activation has been demonstrated by using cancer cells derived from different tumors (e.g., brain, breast, pancreas, and mesothelium). Acting exclusively on TβRI or interfering with type I and II TGF-β receptors, these inhibitors were shown to reduce tumor growth, metastasis, recurrence and angiogenesis in mouse models (Gaspar et al., 2007; Suzuki et al., 2007; Rausch et al., 2009; Zhang et al., 2011).

Given the outstanding results achieved *in vitro* and *in vivo*, kinase inhibitors were also investigated in clinical studies. LY2157299 (or Galunisertib) had its safety demonstrated in a phase I trial with glioblastoma patients (NCT01220271), but the antitumor response was only achieved in 3 of 28 patients. Still, 10 other studies are currently in development or recruiting patients with different types of cancer in advanced stage. LY3200882 is the most recent TGF-β inhibitor in this class and clinical studies (phases I and II) intend to recruit patients to evaluate safety and antitumor activity as single agent or in combination with other chemotherapies (NCT02937272, NCT04031872). Table 4 presents an overview of cancer clinical trials using kinase inhibitors.

**TABLE 4:** Overview of anti-TGF-β therapies based on kinase inhibitors used in cancer clinical trials.

Drug (target)	Clinical trial (phase)	Status	Cancer type	Patients enrolled	Arms	Outcomes
LY2157299 (TβRI)	NCT01220271 (Phases I/II)	Completed	Glioma	75	Phase I LY2157299 (160 mg) + radiotherapy + temozolamide LY2157299 (300 mg) + radiotherapy + temozolamide Phase II LY2157299 (established dose) + radiotherapy + temozolamide Radiotherapy + temozolamide	NA
LY3200882 (TβRI)	NCT02937272 (Phase I)	Active, not recruiting	Solid tumors	223	LY3200882 LY3200882 + LY3300054 LY3200882 + gemcitabine + nab-paclitaxel LY3200882 + cisplatin + radiation	NA
LY3200882 (TβRI)	NCT04031872 (Phases I/II)	Active, not recruiting	Colorectal metastatic cancer	31	Single-arm: LY3200882 + capecitabine	NA

*Cmax, maximum concentration; ORR, objective response rate; PFS, progression-free survival; CBR, clinical benefit rate; CR, complete response; PR, partial response; AUC, area under the curve; SAE, serious adverse event; OS, overall survival; NA, not available.*

As described above, positive results in clinical tests using anti-TGF-β therapies were observed, but they are not common to all patients. Even when interesting outcomes were achieved, they are not satisfactorily distinct from those results reported for current therapies, as would be expected by data obtained *in vitro* and *in vivo*. This highlights a major problem: a gap in the current comprehension about TGF-β activity during cancer progression in human patients. Based on the most recent findings, we argue in next section that important points about TGF-β signaling have or are not being properly considered in preclinical studies. Specifically, we address (i) the dual role of TGF-β signaling in EMT and MET; (ii) TGF-β dynamic signaling; (iii) the functional difference of TGF-β secreted by exosomes; and (iv) the regulatory effects of tumor microenvironment (TME) – particularly by cancer-associated fibroblasts – on TGF-β signaling activities and its functions.

## Controlling Metastasis Critical Steps: the Dual Role of TGF-β

The critical role of TGF-β on EMT, increasing cancer cells migration and invasion *in vitro* (Hao et al., 2019) have been comprehensively established. By using pharmacological inhibitors studies have also demonstrated convincingly that blocking TGF-β signaling represents an effective strategy to impair metastasis *in vivo* (Matthews et al., 2000; Zhong et al., 2010; Biswas et al., 2011; Dutta et al., 2015). Still, few studies consider that TGF-β can exert an important anti-MET activity, avoiding a critical late-stage step in metastasis (Figure 1). Also, cancer patients and animal models differ in a very important point that could be critical to classify TGF-β as friend or foe: the timing at which the treatment is administered.

It is usual to start anti-TGF-β treatment in animal models as soon as the cancer reaches a palpable volume or even earlier. By treating cancer at such an early stage, researchers avoid that malignant cells invade surrounding tissues and progress to vasculature intravasation, inhibiting metastasis and reinforcing the dangerous role of TGF-β in metastasis. Nevertheless, cancers in humans are not always diagnosed at early stages because it takes time until the initial symptoms appear, not mentioning that the TGF-β targeting treatments were often for very late stage cancer patients. Thus, when diagnosis occurs, many tumor cells have already spread and are found in the blood and/or lymph. These CTCs and possible undetectable micrometastases underwent EMT before treatment has started. Therefore, administering anti-TGF-β therapies by this time could block one of the most important molecular pathways that sustain cancer cells mesenchymal phenotype, inducing MET and facilitating the growth of secondary tumors (Figure 1).

Based on their enhanced invasive potential, cancer cells with mesenchymal phenotype usually result in more metastasis than counterparts with epithelial phenotype when implanted in solid tissues. Nevertheless, to represent the CTCs usually observed in cancer patients, these cells should be evaluated after they reach the bloodstream. Indeed, the reduced ability of mesenchymal cells to colonize secondary organs and establish distant metastasis has been described for many cancer types in animal models. In a striking report Biswas et al. (2014) describe that blocking TGF-β activity resulted in different outcomes when employing cancer cells with distinct initial phenotypes. In this study, researchers used a TβRI kinase inhibitor to treat breast cancer cells carrying mesenchymal – or epithelial-like phenotypes. After intracardiac inoculation, epithelial-like cancer cells treated or not with anti-TGF-β metastasized at the same rate, while mesenchymal-like cancer cells responded to TGF-β signaling pathway block by slight increasing the number of lung metastases. By disrupting TGF-β signaling, researchers probably induced MET in these cancer cells, given them more benefits than disadvantages in secondary organ colonization.

Thus, the generally not considered role of TGF-β in promoting anti-MET could actually make this cytokine an interesting friend to block cancer metastasis in advanced tumors that already started to spread. In addition, this potential TGF-β function highlights the relevance of clearly determining which patients should be submitted to TGF-β inhibitors and considering probable poor outcomes for post-operative patients or patients with cancer in advanced stage (Figure 1). A thorough understanding of detailed and exacting roles played by TGF-β at specific cellular stages of cancer metastasis is in urgent need in order to devise an effective, precise anti-TGF-β treatment regimen.

## TGF-β Dynamic Signaling

How treatment schemes for anti-TGF-β therapies are defined? Do these protocols consider natural fluctuations in TGF-β signaling? Similar to observations in many other molecular pathways, TGF-β signaling is also controlled by negative feedbacks, being SMAD7 its best characterized feedback inhibitor. In a simplified model, high levels of stimulus (TGF-β activity) result in increased *SMAD7* expression and TGF-β/SMAD signaling inhibition (SMAD7 activity) that in turn disrupt the initial stimulus (Nakao et al., 1997; Jenkins et al., 2005; Zhang et al., 2007; Khatibi et al., 2017a, b). Therefore, careless administration of TGF-β inhibitors should be expected to result in rapid decrease of TGF-β activity, followed by its pronounced increase when TGF-β receptors are thereafter activated.

Other than proteins, miRNA and lncRNA targeting TGF-β, its receptors or downstream effectors (Hao et al., 2019), TGF-β signaling is also opposed by BMP signaling – commonly associated to a MET-promoter effect – in many metastatic cancers (Gao et al., 2012; Karagiannis et al., 2013; Vollaire et al., 2019). Considering this antagonism, TGF-β pharmacological targeting should increase BMP activity, preventing cancer cell invasion and reducing the risk of metastasis. Nevertheless, two different problems could arise from that strategy. First, as discussed in the previous section, by promoting MET in cancer cells after intravasation/extravasation, BMP signaling pathway would actually contribute to metastatic development. Second, it is not unusual to detect alterations in BMP pathway, being these effects imposed by malignant cells and other elements at the TME. Gremlin 1 (GREM1) is a BMP antagonist that binds to the ligand, preventing its interaction with membrane receptors and activation of downstream signaling pathway. As recently demonstrated by Ren et al. (2019) elevated levels of GREM1 correlate with a poor prognostic for breast cancer patients. Also, the same study showed that GREM1 promotes EMT and invasion of breast cancer cells *in vitro* and it is correlated with higher levels of intravasation and extravasation in a zebrafish model.

Therefore, considering the existence of TGF-β regulatory components, oscillations in TGF-β signaling pathway were mathematically modeled *in silico* and tested *in vitro*. Many groups demonstrated that dynamic changes occur, including fluctuations in levels of SMADs phosphorylation and activity (Zi et al., 2011; Wegner et al., 2012), differential nuclear-cytoplasmic shuttling (Giampieri et al., 2009; Warmflash et al., 2012) and irregular regulatory effects on gene transcription (Giampieri et al., 2009; Zi et al., 2011; Wegner et al., 2012). These alterations are natural results of intracellular homeostasis, but these effects can also be induced by exposure to different ligand concentrations over time (Schneider et al., 2012; Warmflash et al., 2012; Wegner et al., 2012; Wang et al., 2014). It is reasonable to assume that metastatic cells traveling through different tissues on their way to distant organs are not submitted to a homogeneous environment. Otherwise, malignant cells are likely to be subjected to other cell types with heterogeneous TGF-β secretion potentials, what is not commonly reproduced *in vitro* and could result in different states of cancer cell activation. Sorre et al. (2014) studying the influence of this cytokine on the development of *Xenopus* embryos showed that a pulsed stimulus is more effective than a constant elevation in TGF-β concentrations to promote signaling activation. Moreover, Nicolás and Hill (2003) analyzing the tumor suppressive role of TGF-β reported that the resistance to TGF-β-induced growth arrest exhibited by some pancreatic cancer cell lines derive from their ability to rapidly export R-SMADs to cytoplasm, while counterparts sensitive to TGF-β retain nuclear SMADs for longer periods.

However, it should not be expected that all cells in a population (i.e., malignant tumor) present similar responsiveness to TGF-β – or at least not in a synchronized pattern. Evaluating cancer cell migration, Luwor et al. (2015) demonstrated an interesting difference exerted by the dynamics of TGF-β among cells populations *in vitro*. Even though SMAD3 activity was enhanced in migratory cells compared to non-migratory cells, the behavior of migratory cells was uneven. Three subpopulations were classified among these migratory cells, but surprisingly, cells with higher SMAD3 activity moved smaller distances than migratory cells with low or medium SMAD3 activity. Interestingly, this heterogeneous pattern of TGF-β activity in different cells from the same population was also described *in vivo* by Giampieri et al. (2009). Importantly, they demonstrated that TGF-β signaling activity is not sustained during all metastatic steps, and while SMAD2 nuclear localization and SMAD3 activity were detectable in migrating cells, these results were not present in cancer cells in lymph node metastases.

**FIGURE 3:**
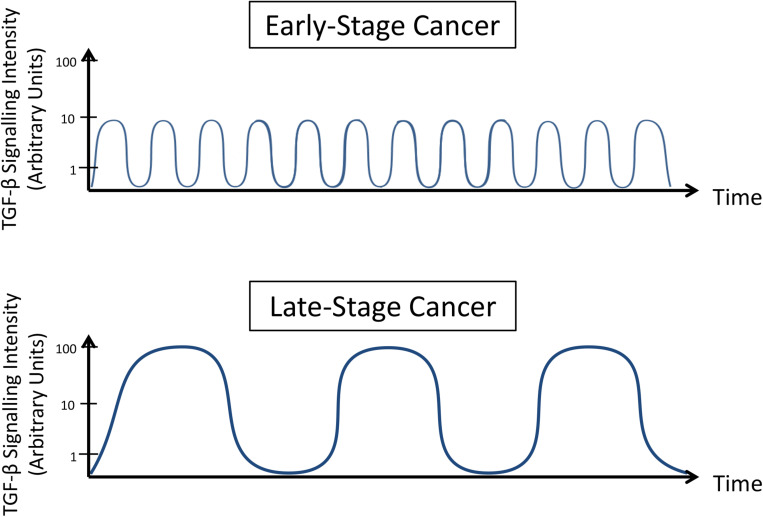
TGF-β signaling dynamics change during cancer progression. Late-stage cancer cells present increased intensity and reduced frequency oscillation in TGF-β signaling pathway activity compared to early stage cancer cells.

Overall, these results demonstrate that TGF-β signaling is dynamic. Two main mechanisms explain this observation: (i) this molecular pathway is transiently regulated by a negative feedback involving molecules (proteins or RNAs) that present direct interaction with TGF-β or its effectors; and (ii) opposite signaling pathways can inhibit TGF-β signaling for prolonged times and avoid its control on cell phenotype. Understanding how these processes occur in different contexts and reproduce this balance in pre-clinical models will help to establish a better treatment scheme in clinical trials at which anti-TGF-β therapies are carefully administered to prevent amplified TGF-β activation (Figure 3), as such normalizing its signaling.

## Tumor Microenvironment Regulates TGF-β Signaling

The immunosuppressive role of TGF-β has been explored in many diseases including cancer and the combination of TGF-β inhibitors and immunotherapies is suggested as an alternative to improve the antitumor effect of immune cells. Still, this scenario considers components of TME – especially immune cells – as targets rather than sources of TGF-β secretion. It has been demonstrated, for instance, that cells such as myeloid-derived suppressor cells (MDSCs) secrete TGF-β and contribute to cancer progression (Yang et al., 2008; Biswas et al., 2019). Still, other cell types (e.g., endothelial cells and pericytes) already described as important during metastasis could also be involved in this process, but their contribution to carcinogenesis based on TGF-β secretion still not properly explored (Flaumenhaft et al., 1993; Ma et al., 2007; Colak and Ten Dijke, 2017). Therefore, despite cancer cells, little is known about TGF-β secretion by TME cells.

Considering the interference of cancer-associated fibroblasts (CAFs) in multiple processes during carcinogenesis (e.g., ECM remodeling, angiogenesis, bioenergetics, cancer cells stemness, response to therapies and immune surveillance), their ability to promote TGF-β-mediated EMT, invasion and metastasis started to be evaluated. Yu et al. (2014) demonstrated that primary breast CAFs-conditioned medium induce EMT markers (e.g., vimentin upregulation and E-cadherin downregulation) in breast cancer cell lines, promoting migration and invasion *in vitro* that could be partially blocked by using an anti-TGF-β antibody or a TβRI kinase inhibitor. Similar results were obtained by Nagura et al. (2015) working with uterine cervical squamous cell carcinoma cells. Surprisingly, they also showed that an exclusive detection of phosphorylated SMAD3 at tumor boundaries were preferentially detected in samples from uterine cancer patients diagnosed with lymph node metastasis, suggesting that the activation of TGF-β signaling pathway in malignant cells is induced by tumor stroma. Liu et al. (2016a, b) using primary fibroblasts isolated from hepatocellular carcinoma patients confirmed the relevance of TME cells signaling on EMT, migration and invasion *in vitro* and metastasis *in vivo* as a result of TGF-β signaling activation.

As discussed before, TGF-β and BMP signaling pathways are commonly describe as opposite molecular pathways, but the consequent effects are not limited to cancer cells. Gao et al. (2012) showed that lung stromal cells restrict the growth of metastasis by secreting high levels of active BMP. Interestingly, the ability of breast cancer cells to overcome this anti metastatic mechanism was related to secretion of COCO (DAND5) and consequent inhibition of BMP signaling. Ren et al. (2019), in the same study mentioned before, described that TGF-β-induced CAFs activation results in GREM1 (BMP inhibitor) secretion *in vitro*. This suggestive feed forward loop between CAFs and malignant cells was further reinforced by results showing that GREM1 expression is restricted to tumor stroma in breast cancer patients.

**FIGURE 4:**
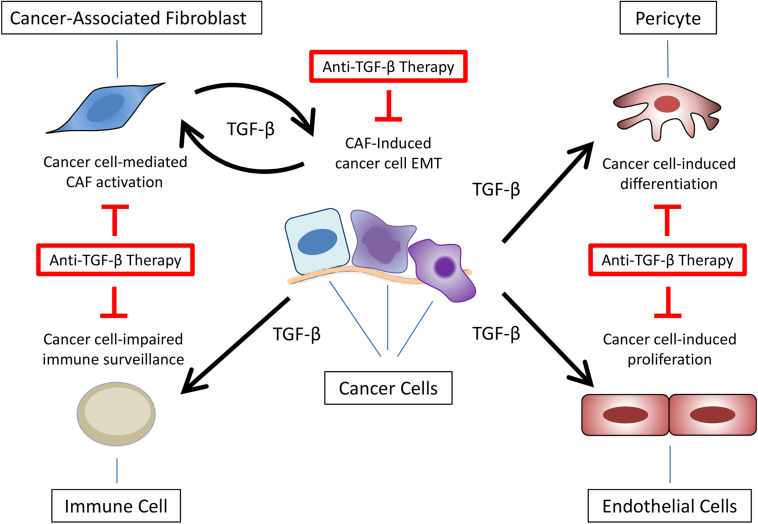
Anti-TGF-β therapies targeting multiple components of tumor microenvironment. Besides epithelial-mesenchymal transition (EMT) in cancer cells, TGF-β secretion at the tumor microenvironment promotes cancer-associated fibroblast (CAF) activation, impaired immune surveillance, pericytes differentiation, and endothelial cell proliferation.

Overall, these data highlight the importance of the microenvironment surrounding cancer cells – especially CAFs – secreting or controlling the secretion of TGF-β. Nevertheless, the presence of CAFs in animal models exploring the response to anti-TGF-β therapies is not commonly observed. Thus, together with the points discussed in the previous sections, ignoring the crosstalk between cancer cells and TME components – especially CAFs – could hamper the translation of preclinical results to clinical trials using TGF-β signaling pathway inhibitors, explaining the poor and inconsistent outcomes observed in cancer patients (Figure 4), highlighting the need for developing mouse tumor models containing TME or at least CAFs.

## Exosomes as a Mechanism of TGF-β Secretion and Signaling Amplification

Exosomes are nanosized extracellular vesicles with a diameter ranging from 30 to 100 nm. The role of exosomes on cell communication relies on their ability to transport different types of cargo allowing cell-cell interaction and autocrine or paracrine signaling. Nucleic acids, lipids and many proteins were already described among exosomes cargo, but their specific mechanism of sorting into endosomes still poorly understood (Escrevente et al., 2011; Christianson et al., 2013; Li et al., 2017). Interestingly, even though TGF-β receptors traditionally localize at the plasma membrane, they present sequences used by cell machinery as signals for internalization (Di Guglielmo et al., 2003; Clement et al., 2013). Indeed, it has been shown that these receptors can be directed to early endosomes, activating the SMAD-dependent pathway before being recycled back to the cell surface (Di Guglielmo et al., 2003; Clement et al., 2013). Thus, considering the role of endosomes as precursors of exosomes, the secretion of TGF-β by these extracellular vesicles became a suggestive possibility.

In fact, some studies have showed the secretion of exosomal TGF-β by cancer cells and their interaction with other TME components. For instance, Rong et al. (2016) demonstrated *in vitro* that T cells treated with exosomes derived from breast cancer cell lines exhibit reduced proliferation through a TGF-β-dependent mechanism that could be only partially reverted by treatment with anti-TGF-β antibodies. Furthermore, after show that stage III-IV renal carcinoma patients present higher levels of exosomal TGF-β than patients in stages I-II, Xia et al. (2017) treated NK cells with tumor-derived exosomes and reported a decrease in their cytotoxicity. Also, using 786-O renal adenocarcinoma cells as a model, the same study demonstrated that TGF-β secreted in exosomes is more efficient to reduce NK cytotoxicity than its free-ligand form. In gastric cancer, Yen et al. (2017) showed that exosomal TGF-β isolated from peripheral blood is elevated in patients with lymph node metastasis and positively correlates with increased levels of T regulatory lymphocytes.

Reinforcing the role of exosomes in paracrine signaling, Webber et al. (2010) reported that primary fibroblasts are activated after treatment with exosomal TGF-β secreted by cancer cells. Interestingly, although most TGF-β in these exosomes was present in the latent form, cells stimulated with exosomal TGF-β and TGF-β in its free-ligand form exhibited similar levels of SMAD3 activity. Still, in the opposite direction of this crosstalk, Li et al. (2017) demonstrated that CAFs can also secrete exosomal TGF-β. Even more, exosomal TGF-β was show to promote SMAD2/3 phosphorylation in ovarian cancer cells, decreasing their levels of E-cadherin and increasing vimentin expression. As a result of exposure to exosomal TGF-β secreted by CAFs, cancer cells presented enhanced migration and invasion *in vitro* and increased tumor growth *in vivo*.

While studies exploring the mechanisms of carcinogenesis have mostly focused on exosomes as the main type of extracellular vesicles mediating TGF-β transport as cargo, TGF-β secretion in microvesicles/ectosomes have also been reported in other contexts such as in immunology and infectious diseases (Cestari et al., 2012; Sadallah et al., 2014, 2016). For instance, Cestari et al. (2012) showed that *Trypanosoma cruzi* infection induces the release of microvesicles (MVs) enriched in TGF-β from blood cells. These MVs associate with the parasite surface, increasing *T. cruzi* invasion into host cells and escape from complement system. Other than that, two studies from Sadallah and collaborators demonstrated that platelet-derived MVs enriched in TGF-β promote immunosuppression by both promoting CD4^+^ T cells differentiation toward a Treg phenotype (Sadallah et al., 2014) and decreasing NK cells activity (Sadallah et al., 2014).

Thus, the extracellular vesicles could work as an alternative mechanism to secrete TGF-β, and particularly in the context of cancer progression, promoting the crosstalk between TME cells and even amplifying TGF-β signaling activation. Furthermore, it is possible that extracellular vesicles prevent the interaction between TGF-β and ligand traps in a mechanism similar to what was shown for exosomes transporting PD-L1 and impairing anti-PD-L1 antibodies activity (Poggio et al., 2019). Also, the uptaking of extracellular vesicles enriched in TGF-β could activate TGF-β signaling from the cytoplasm. As such, the dynamics of extracellular vesicles trafficking in different cell types likely to influence the extent of downstream effects. As different mechanisms to block exosomes secretion and uptake have been tested *in vitro* (Ostrowski et al., 2010; Christianson et al., 2013), a combinatory approach targeting exosomes and TGF-β signaling simultaneously should be evaluated in order to block both TGF-β forms of secretion – as a free-ligand and as extracellular vesicles cargo.

## Other Perspectives

As discussed in the previous sections, anti-TGF-β therapies that successfully inhibit cancer cells EMT, migration, invasion and metastasis in pre-clinical models have faced multiple problems when used to treat cancer patients in clinical trials. However, the combination of these same drugs with immune checkpoint inhibitors has recently emerged as a highly promising approach that can lead to a prolonged anti-cancer response.

The immunosuppressive activity played by TGF-β critically impacts the activity of different immune cell types (Yang et al., 2010). More specifically, TGF-β is shown to reduce the proliferation (Rong et al., 2016) and activity (Thomas and Massagué, 2005) of cytotoxic T cells while induces the differentiation of CD4^+^ cells toward a Treg phenotype (Chen et al., 2003; Ghiringhelli et al., 2005). Based on this premise, for instance, Dodagatta-Marri et al. (2019) have shown that outcomes obtained with an anti-PD-1 antibody can be improved by combining it with an anti-TGF-β neutralizing antibody treatment in xenograft models of skin cancer. In this study, while the outcome obtained with the anti-PD-1 therapy was limited to a partial regression and correlated with an increased CD4^+^ Treg/CD4^+^ Th cells ratio, a complete tumor regression was achieved by the synergistic response when anti-PD-1 and anti-TGF-β antibodies were combined. Results from Sow et al. (2019) have corroborated this pattern by demonstrating that the combination of an anti-PD-L1 antibody with a TβRI kinase inhibitor lead to increased survival in the highly immunogenic mouse MC38 colon adenocarcinoma model. Still, because the same combinatorial approach did not lead to similar outcomes in the poorly immunogenic mouse KPC1 pancreatic cancer model, these researches also suggested that combining anti-TGF-β therapies with immune checkpoint inhibitors may be beneficial only for certain types of cancer, highlighting the relevance of an appropriate selection of patients to undergo this therapeutic strategy. In addition, it is noteworthy that the role played by TGF-β in blocking the infiltration of cytotoxic T cells into the cancer mass may not necessarily be induced by cancer cells. In fact, Mariathasan et al. (2018) and Tauriello et al. (2018) demonstrated that the TGF-β-mediated immune exclusion is a response triggered during cancer progression by non-cancer cells from the tumor stroma, particularly by CAFs.

Overall, considering the studies highlighted in this section and the evidences from the use of bifunctional antibodies that simultaneously target TGF-β and PD-1/PD-L1 (as presented in section “Interfering With Ligand-Receptor Interactions.” Interfering with ligand-receptor interactions), it is suggestive that the poor outcomes obtained with anti-TGF-β therapies in clinical trials may be improved by their combination with other therapies, particularly with immune checkpoint inhibitors. In this context, experimental studies regarding the immunosuppressive activity played by TGF-β should be expanded and their results compared between “cold tumors” and “hot tumors” in order to obtain a better understanding about the use of TGF-β pharmacological inhibitors to overcome the immune exclusion that is common to different types of cancer. While the use of immune checkpoint inhibitors alone may favor the cytolytic activity of immune cells that are present within the cancer mass, their combination with TGF-β pharmacological inhibitors may increase the infiltration of these cells in “cold tumors.” More details about the synergism between TGF-β pharmacological inhibitors and immune checkpoint inhibitors has been reviewed and discussed by others regarding its use in pre-clinical models and clinical trials (Ganesh and Massagué, 2018; Bai et al., 2019; Groeneveldt et al., 2020; Lind et al., 2020).

Given the occasional but serious side effect of anti-PD1/PD-L1 therapies on heart (Bajwa et al., 2019) and TGF-β’s important role played in heart development and homeostasis (Dickson et al., 1995; Stenvers et al., 2003; Anderton et al., 2011), the challenges for the combined or bifunctional antibody therapies are to minimize the potential fatal side effect.

## Concluding Remarks

In the past decade(s) many studies have been devoted to delineate the dynamic role of TGF-β signaling in the multistep process of metastasis. While substantial insights were obtained, new layers of complexity and regulation continue to be discovered. Its potent pro-oncogenic activities have been targeted using a scale of selective pharmacological inhibitors. Reported results in cancer models have been very promising. Yet, outcomes observed in more than 20 cancer clinical trials using anti-TGF-β therapies lack consistency and fail to recapitulate the preclinical data, raising questions about what is missing when translating these strategies from bench-to-bedside.

TGF-β function in cancer cell invasion and metastasis is pleiotropic and dynamically controlled. These critical aspects are somewhat overlooked when TGF-β targeting agents are applied to cancer patients. The administration of these drugs to post-operative patients or patients with advanced cancers could increase the seeding of CTCs and the growth of metastatic lesions by blocking its anti-MET activity and its tumor-suppressor function. Also, affected by the activity of antagonist molecular pathways, induced by surrounding elements in the TME and other cancer cells, the dynamics of TGF-β signaling might change from normal to malignant cells (and even from early to late-stage cancer cells) thereby influencing how different patients (or different cells in the same patient) respond to the therapy. Finally, the presence of TGF-β in exosomes could make it inaccessible from antibodies and ligand traps, limiting the effectiveness of these intervention strategies to the freely soluble ligand form of this cytokine. New *in vitro* and *in vivo* models exploiting these points will deepen our knowledge about the real complexity of TGF-β signaling in carcinogenesis. To do so, sensitive technologies such as single cell signaling and live signaling *in vitro* and *in vivo* are to be employed (Zhou et al., 2014; Luwor et al., 2015; Chen et al., 2018) and further developed. Moreover, clinical trials combining anti-TGF-β therapies to other target therapies such as immune checkpoint inhibitors, or strategies allowing a more spatio-temporal controlled intervention using nanocarriers, may allow for an improved treatment and perhaps even cure of cancer patients.

## Author Contributions

H-JZ: concept formation. AT: writing and original draft preparation. PD and H-JZ: editing and revision. All authors contributed to the article and approved the submitted version.

## Conflict of Interest

The authors declare that the research was conducted in the absence of any commercial or financial relationships that could be construed as a potential conflict of interest.

## Abbreviations

ASO: antisense oligonucleotide
BMP: bone morphogenetic protein
CAF: cancer-associated fibroblast
Co-SMAD: common SMAD
CTC: circulating tumor cell
ECM: extracellular matrix
EMT: epithelial-mesenchymal transition
GREM1: gremlin 1
I-SMAD: inhibitor SMAD
LAP: latency-associated peptide
LLC: large latent complex
LTBP: latent TGF-β binding protein
MET: mesenchymal-epithelial transition
R-SMAD: receptor SMAD
sRII/III: soluble T β RII/III
TGF-β: transforming growth factor-beta
TME: tumor microenvironment
T β RI/II/III: TGF-β receptor type I/II/II.

## References

[B1] Ahmadi A. Najafi M. Farhood B. Mortezaee K. (2019). Transforming growth factor-β signaling: tumorigenesis and targeting for cancer therapy. *J. Cell Physiol.* 234 12173–12187. 10.1002/jcp.27955 30537043 PMC13484046

[B2] Anderton M. J. Mellor H. R. Bell A. Sadler C. Pass M. Powell S. (2011). Induction of heart valve lesions by small-molecule ALK5 inhibitors. *Toxicol. Pathol.* 39 916–924. 10.1177/0192623311416259 21859884

[B3] Andres J. L. DeFalcis D. Noda M. Massagué J. (1992). Binding of two growth factor families to separate domains of the proteoglycan betaglycan. *J. Biol. Chem.* 267 5927–5930. 1556106

[B4] Anzano M. A. Roberts A. B. Smith J. M. Lamb L. C. Sporn M. B. (1982). Purification by reverse-phase high-performance liquid chromatography of an epidermal growth factor-dependent transforming growth factor. *Anal. Biochem.* 125 217–224. 10.1016/0003-2697(82)90405-5 6983313

[B5] Arteaga C. L. Hurd S. D. Winnier A. R. Johnson M. D. Fendly B. M. Forbes J. T. (1993). Anti-transforming growth factor (TGF)-beta antibodies inhibit breast cancer cell tumorigenicity and increase mouse spleen natural killer cell activity. Implications for a possible role of tumor cell/host TGF-beta interactions in human breast cancer progression. *J. Clin. Invest.* 92 2569–2576. 10.1172/jci116871 7504687 PMC288452

[B6] Australian Institute of Health and Welfare [AIHW] (2019). *Cancer in Australia 2019. Cancer Series No.119. Cat. No. CAN 123.* Canberra: AIHW.

[B7] Bai X. Yi M. Jiao Y. Chu Q. Wu K. (2019). Blocking TGF-β signaling to enhance the efficacy of immune checkpoint inhibitor. *Onco. Targets Ther.* 12 9527–9538. 10.2147/OTT.S224013 31807028 PMC6857659

[B8] Bajwa R. Cheema A. Khan T. Amirpour A. Paul A. Chaughtai S. (2019). Adverse effects of immune checkpoint inhibitors (Programmed Death-1 Inhibitors and Cytotoxic T-Lymphocyte-Associated Protein-4 Inhibitors): results of a retrospective study. *J. Clin. Med. Res.* 11 225–236. 10.14740/jocmr3750 30937112 PMC6436564

[B9] Bandyopadhyay A. Zhu Y. Cibull M. L. Bao L. Chen C. Sun L. (1999). A soluble transforming growth factor β type III receptor suppresses tumorigenicity and metastasis of human breast cancer MDA-MB-231 cells. *Cancer Res.* 59 5041–5046. 10519421

[B10] Bhola N. E. Balko J. M. Dugger T. C. Kuba M. G. Sánchez V. Sanders M. (2013). TGF-β inhibition enhances chemotherapy action against triple-negative breast cancer. *J. Clin. Invest.* 123 1348–1358. 10.1172/JCI65416 23391723 PMC3582135

[B11] Biswas S. Mandal G. Chowdhury S. Purohit S. Payne K. K. Anadon C. (2019). Exosomes produced by mesenchymal stem cells drive differentiation of myeloid cells into immunosuppressive M2-polarized macrophages in breast cancer. *J. Immunol.* 203 3447–3460. 10.4049/jimmunol.1900692 31704881 PMC6994919

[B12] Biswas S. Nyman J. S. Alvarez J. Chakrabarti A. Ayres A. Sterling J. (2011). Anti-transforming growth factor β antibody treatment rescues bone loss and prevents breast cancer metastasis to bone. *PLoS One* 6:e27090. 10.1371/journal.pone.0027090 22096521 PMC3214031

[B13] Biswas S. Trobridge P. Romero-Gallo J. Billheimer D. Myeroff L. L. Willson J. K. (2008). Mutational inactivation of TGFBR2 in microsatellite unstable colon cancer arises from the cooperation of genomic instability and the clonal outgrowth of transforming growth factor beta resistant cells. *Genes Chromosomes Cancer* 47 95–106. 10.1002/gcc.20511 17985359

[B14] Biswas T. Gu X. Yang J. Ellies L. G. Sun L. Z. (2014). Attenuation of TGF-β signaling supports tumor progression of a mesenchymal-like mammary tumor cell line in a syngeneic murine model. *Cancer Lett.* 346 129–138. 10.1016/j.canlet.2013.12.018 24368187 PMC3947668

[B15] Bray F. Ferlay J. Soerjomataram I. Siegel R. L. Torre L. A. Jemal A. (2018). Global cancer statistics 2018: GLOBOCAN estimates of incidence and mortality worldwide for 36 cancers in 185 countries. *CA Cancer J. Clin.* 68 394–424. 10.3322/caac.21492 30207593

[B16] Cestari I. Ansa-Addo E. Deolindo P. Inal J. M. Ramirez M. I. (2012). Trypanosoma cruzi immune evasion mediated by host cell-derived microvesicles. *J. Immunol.* 188 1942–1952. 10.4049/jimmunol.1102053 22262654

[B17] Chaffer C. L. Brennan J. P. Slavin J. L. Blick T. Thompson E. W. Williams E. D. (2006). Mesenchymal-to-epithelial transition facilitates bladder cancer metastasis: role of fibroblast growth factor receptor-2. *Cancer Res.* 66 11271–11278. 10.1158/0008-5472.can-06-2044 17145872

[B18] Chen H. Ware T. M. B. Iaria J. Zhu H. J. (2018). Live cell imaging of the TGF-β/Smad3 signaling pathway *in vitro* and *in vivo* using an adenovirus reporter system. *J. Vis. Exp.* 57926. 10.3791/57926 30102266 PMC6126585

[B19] Chen W. Jin W. Hardegen N. Lei K. J. Li L. Marinos N. (2003). Conversion of peripheral CD4+CD25- naive T cells to CD4+CD25+ regulatory T cells by TGF-beta induction of transcription factor Foxp3. *J. Exp. Med.* 198 1875–1886. 10.1084/jem.20030152 14676299 PMC2194145

[B20] Christianson H. C. Svensson K. J. van Kuppevelt T. H. Li J.-P. Belting M. (2013). Cancer cell exosomes depend on cell-surface heparan sulfate proteoglycans for their internalization and functional activity. *Proc. Natl. Acad. Sci. U.S.A.* 110 17380–17385. 10.1073/pnas.1304266110 24101524 PMC3808637

[B21] Clement C. A. Ajbro K. D. Koefoed K. Vestergaard M. L. Veland I. R. Henriques (2013). TGF-β signaling is associated with endocytosis at the pocket region of the primary cilium. *Cell Rep.* 3 1806–1814. 10.1016/j.celrep.2013.05.020 23746451

[B22] Colak S. Ten Dijke P. (2017). Targeting TGF-β signaling in cancer. *Trends Cancer* 3 56–71. 10.1016/j.trecan.2016.11.008 28718426

[B23] Cooley J. R. Yatskievych T. A. Antin P. B. (2014). Embryonic expression of the transforming growth factor beta ligand and receptor genes in chicken. *Dev. Dyn.* 243 497–508. 10.1002/dvdy.24085 24166734 PMC3969743

[B24] de Jonge R. R. Garrigue-Antar L. Vellucci V. F. Reiss M. (1997). Frequent inactivation of the transforming growth factor beta type II receptor in small-cell lung carcinoma cells. *Oncol. Res.* 9 89–98. 9167190

[B25] de Larco J. E. Todaro G. J. (1978). Growth factors from murine sarcoma virus-transformed cells. *Proc Natl Acad Sci U.S.A.* 75 4001–4005. 10.1073/pnas.75.8.4001 211512 PMC392918

[B26] Denney L. Byrne A. J. Shea T. J. Buckley J. S. Pease J. E. Herledan G. M. (2015). Pulmonary epithelial cell-derived cytokine TGF-β1 is a critical cofactor for enhanced innate lymphoid cell function. *Immunity* 43 945–958. 10.1016/j.immuni.2015.10.012 26588780 PMC4658339

[B27] Di Guglielmo G. M. Le Roy C. Goodfellow A. F. Wrana J. L. (2003). Distinct endocytic pathways regulate TGF-beta receptor signalling and turnover. *Nat. Cell Biol.* 5 410–421. 10.1038/ncb975 12717440

[B28] Dickson M. C. Martin J. S. Cousins F. M. Kulkarni A. B. Karlsson S. Akhurst R. J. (1995). Defective haematopoiesis and vasculogenesis in transforming growth factor-β 1 knock out mice. *Development* 121 1845–1854. 7600998 10.1242/dev.121.6.1845

[B29] Dodagatta-Marri E. Meyer D. S. Reeves M. Q. Paniagua R. To M. D. Binnewies M. (2019). α-PD-1 therapy elevates Treg/Th balance and increases tumor cell pSmad3 that are both targeted by α-TGFβ antibody to promote durable rejection and immunity in squamous cell carcinomas. *J. Immunother. Cancer* 7:62. 10.1186/s40425-018-0493-9 30832732 PMC6399967

[B30] Dutta A. Li J. Fedele C. Sayeed A. Singh A. Violette S. M. (2015). αvβ6 integrin is required for TGFβ1-mediated matrix metalloproteinase2 expression. *Biochem. J.* 15 525–536. 10.1042/BJ20140698 25558779 PMC4363118

[B31] Escrevente C. Keller S. Altevogt P. Costa J. (2011). Interaction and uptake of exosomes by ovarian cancer cells. *BMC Cancer* 11:108. 10.1186/1471-2407-11-108 21439085 PMC3072949

[B32] Ferlay J. Colombet M. Soerjomataram I. Mathers C. Parkin D. M. Piñeros M. (2019). Estimating the global cancer incidence and mortality in 2018: GLOBOCAN sources and methods. *Int. J. Cancer* 144 1941–1953. 10.1002/ijc.31937 30350310

[B33] Fitzpatrick D. R. Bielefeldt-Ohmann H. Himbeck R. P. Jarnicki A. G. Marzo A. L. Robinson B. W. (1994). Transforming growth factor-beta: antisense RNA-mediated inhibition affects anchorage-independent growth, tumorigenicity and tumor-infiltrating T-cells in malignant mesothelioma. *Growth Fact.* 11 29–44. 10.3109/08977199409015049 7833058

[B34] Flaumenhaft R. Abe M. Sato Y. Miyazono K. Harpel J. Heldin C. H. (1993). Role of the latent TGF-beta binding protein in the activation of latent TGF-beta by co-cultures of endothelial and smooth muscle cells. *J. Cell Biol.* 120 995–1002. 10.1083/jcb.120.4.995 8432736 PMC2200078

[B35] Fleming N. I. Jorissen R. N. Mouradov D. Christie M. Sakthianandeswaren A. Palmieri M. (2013). SMAD2, SMAD3 and SMAD4 mutations in colorectal cancer. *Cancer Res.* 73 725–735. 10.1158/0008-5472.CAN-12-2706 23139211

[B36] Ganapathy V. Ge R. Grazioli A. Xie W. Banach-Petrosky W. Kang Y. (2010). Targeting the Transforming Growth Factor-β pathway inhibits human basal-like breast cancer metastasis. *Mol. Cancer* 9 122–137. 10.1186/1476-4598-9-122 20504320 PMC2890606

[B37] Ganesh K. Massagué J. (2018). TGF-β inhibition and immunotherapy: checkmate. *Immunity* 48 626–628. 10.1016/j.immuni.2018.03.037 29669246 PMC6347120

[B38] Gao H. Chakraborty G. Lee-Lim A. P. Mo Q. Decker M. Vonica A. (2012). The BMP inhibitor Coco reactivates breast cancer cells at lung metastatic sites. *Cell* 150 764–779. 10.1016/j.cell.2012.06.035 22901808 PMC3711709

[B39] Gaspar N. J. Li L. Kapoun A. M. Medicherla S. Reddy M. Li G. (2007). Inhibition of transforming growth factor beta signaling reduces pancreatic adenocarcinoma growth and invasiveness. *Mol. Pharmacol.* 72 152–161. 10.1124/mol.106.029025 17400764

[B40] Ghiringhelli F. Ménard C. Terme M. Flament C. Taieb J. Chaput N. (2005). CD4+CD25+ regulatory T cells inhibit natural killer cell functions in a transforming growth factor-beta-dependent manner. *J. Exp. Med.* 202 1075–1085. 10.1084/jem.20051511 16230475 PMC2213209

[B41] Giampieri S. Manning C. Hooper S. Jones L. Hill C. S. Sahai E. (2009). Localized and reversible TGFbeta signalling switches breast cancer cells from cohesive to single cell motility. *Nat. Cell Biol.* 11 1287–1296. 10.1038/ncb1973 19838175 PMC2773241

[B42] Gladilin E. Ohse S. Boerries M. Busch H. Xu C. Schneider M. (2019). TGFβ-induced cytoskeletal remodeling mediates elevation of cell stiffness and invasiveness in NSCLC. *Sci. Rep.* 21 7667–7678. 10.1038/s41598-019-43409-x 31113982 PMC6529472

[B43] Grenga I. Donahue R. N. Gargulak M. L. Lepone L. M. Roselli M. Bilusic M. (2018). Anti-PD-L1/TGFβR2 (M7824) fusion protein induces immunogenic modulation of human urothelial carcinoma cell lines, rendering them more susceptible to immune-mediated recognition and lysis. *Urol. Oncol.* 36 93.e1–93.e11. 10.1016/j.urolonc.2017.09.027 29103968 PMC5835162

[B44] Groeneveldt C. van Hall T. van der Burg S. H. Ten Dijke P. van Montfoort N. (2020). Immunotherapeutic potential of TGF-β inhibition and oncolytic viruses. *Trends Immunol.* 41 406–420. 10.1016/j.it.2020.03.003 32223932

[B45] Hachim M. Y. Hachim I. Y. Dai M. Ali S. Lebrun J. J. (2018). Differential expression of TGFβ isoforms in breast cancer highlights different roles during breast cancer progression. *Tumour Biol.* 40:1010428317748254. 10.1177/1010428317748254 29320969

[B46] Hackett P. B. Varmus H. E. Bishop J. M. (1981). The genesis of Rous sarcoma virus messenger RNAs. *Virology* 112 714–728. 10.1016/0042-6822(81)90316-0 6266148

[B47] Hahn S. A. Schutte M. Hoque A. T. Moskaluk C. A. da Costa L. T. Rozenblum E. (1996). DPC4, a candidate tumor suppressor gene at human chromosome 18q21.1. *Science* 271 350–353. 10.1126/science.271.5247.350 8553070

[B48] Hanahan D. Weinberg R. A. (2000). The hallmarks of cancer. *Cell* 7 57–70. 10.1016/s0092-8674(00)81683-9 10647931

[B49] Hao Y. Baker D. Ten Dijke P. (2019). TGF-β-mediated epithelial-mesenchymal transition and cancer metastasis. *Int J. Mol. Sci.* 20:2767. 10.3390/ijms20112767 31195692 PMC6600375

[B50] Itoh F. Itoh S. Carvalho R. L. Adachi T. Ema M. Goumans M. J. (2009). Poor vessel formation in embryos from knock-in mice expressing ALK5 with L45 loop mutation defective in Smad activation. *Lab. Invest.* 89 800–810. 10.1038/labinvest.2009.37 19398960

[B51] Itoh S. Thorikay M. Kowanetz M. Moustakas A. Itoh F. Heldin C. H. (2003). Elucidation of Smad requirement in transforming growth factor-β type I receptor-induced responses. *J. Biol. Chem.* 278 3751–3761. 10.1074/jbc.m208258200 12446693

[B52] Jenkins B. J. Grail D. Nheu T. Najdovska M. Wang B. Waring P. (2005). Hyperactivation of Stat3 in gp130 mutant mice promotes gastric hyperproliferation and desensitizes TGF-beta signaling. *Nat. Med.* 11 845–852. 10.1038/nm1282 16041381

[B53] Karagiannis G. S. Berk A. Dimitromanolakis A. Diamandis E. P. (2013). Enrichment map profiling of the cancer invasion front suggests regulation of colorectal cancer progression by the bone morphogenetic protein antagonist, gremlin-1. *Mol. Oncol.* 7 826–839. 10.1016/j.molonc.2013.04.002 23659962 PMC5528431

[B54] Kattla J. J. Carew R. M. Heljic M. Godson C. Brazil D. P. (2008). Protein kinase B/Akt activity is involved in renal TGF-β1-driven epithelial-mesenchymal transition in vitro and in vivo. *Am. J. Physiol. Renal Physiol.* 295 F215–F225. 10.1152/ajprenal.00548.2007 18495798 PMC2494512

[B55] Khatibi S. Babon J. Wagner J. Manton J. H. Tan C. W. Zhu H. J. (2017a). TGF-β and IL-6 family signalling crosstalk: an integrated model. *Growth Fact.* 35 100–124. 10.1080/08977194.2017.1363746 28948853

[B56] Khatibi S. Zhu H. J. Wagner J. Tan C. W. Manton J. H. Burgess A. W. (2017b). Mathematical model of TGF-β signalling: feedback coupling is consistent with signal switching. *BMC Syst. Biol.* 11:48. 10.1186/s12918-017-0421-5 28407804 PMC5390422

[B57] Kim M. Y. Oskarsson T. Acharyya S. Nguyen D. X. Zhang X. H. Norton L. (2009). Tumor self-seeding by circulating cancer cells. *Cell* 139 1315–1326. 10.1016/j.cell.2009.11.025 20064377 PMC2810531

[B58] Kim Y. E. Won M. Lee S. G. Park C. Song C. H. Kim K. K. (2019). RBM47-regulated alternative splicing of TJP1 promotes actin stress fiber assembly during epithelial-to-mesenchymal transition. *Oncogene* 38 6521–6536. 10.1038/s41388-019-0892-5 31358901

[B59] Lebrun J. J. (2012). The dual role of TGFβ in human cancer: from tumor suppression to cancer metastasis. *ISRN Mol. Biol.* 2012:381428. 10.5402/2012/381428 27340590 PMC4899619

[B60] Levinson A. D. Oppermann H. Levintow L. Varmus H. E. Bishop J. M. (1978). Evidence that the transforming gene of avian sarcoma virus encodes a protein kinase associated with a phosphoprotein. *Cell* 15 561–572. 10.1016/0092-8674(78)90024-7 214242

[B61] Li W. Zhang X. Wang J. Li M. Cao C. Tan J. (2017). TGFβ1 in fibroblasts-derived exosomes promotes epithelial-mesenchymal transition of ovarian cancer cells. *Oncotarget* 8 96035–96047. 10.18632/oncotarget.21635 29221185 PMC5707079

[B62] Lind H. Gameiro S. R. Jochems C. Donahue R. N. Strauss J. Gulley J. L. (2020). Dual targeting of TGF-β and PD-L1 via a bifunctional anti-PD-L1/TGF-βRII agent: status of preclinical and clinical advances. *J. Immunother. Cancer* 8:e000433. 10.1136/jitc-2019-000433 32079617 PMC7057416

[B63] Liu F. L. Mo E. P. Yang L. Du J. Wang H. S. Zhang H. (2016a). Autophagy is involved in TGF-β1-induced protective mechanisms and formation of cancer-associated fibroblasts phenotype in tumor microenvironment. *Oncotarget* 7 4122–4141. 10.18632/oncotarget.6702 26716641 PMC4826194

[B64] Liu J. Chen S. Wang W. Ning B. F. Chen F. Shen W. (2016b). Cancer-associated fibroblasts promote hepatocellular carcinoma metastasis through chemokine-activated hedgehog and TGF-β pathways. *Cancer Lett.* 379 49–59. 10.1016/j.canlet.2016.05.022 27216982

[B65] Liu S. Iaria J. Simpson R. J. Zhu H. J. (2018). Ras enhances TGF-β signaling by decreasing cellular protein levels of its type II receptor negative regulator SPSB1. *Cell Commun. Signal.* 16 10–24. 10.1186/s12964-018-0223-4 29534718 PMC5850916

[B66] Liu Y. Shang D. (2020). Transforming growth factor-β1 enhances proliferative and metastatic potential by up-regulating lymphoid enhancer-binding factor 1/integrin αMβ2 in human renal cell carcinoma. *Mol. Cell Biochem.* 465 165–174. 10.1007/s11010-019-03676-8 31848806

[B67] López-Casillas F. Wrana J. L. Massagué J. (1993). Betaglycan presents ligand to the TGF β signaling receptor. *Cell* 73 1435–1444. 10.1016/0092-8674(93)90368-z 8391934

[B68] Luwor R. B. Hakmana D. Iaria J. Nheu T. V. Simpson R. J. Zhu H. J. (2015). Single live cell TGF-β signalling imaging: breast cancer cell motility and migration is driven by sub-populations of cells with dynamic TGF-β-Smad3 activity. *Mol. Cancer* 4 50–57. 10.1186/s12943-015-0309-1 25744371 PMC4343191

[B69] Ma J. Wang Q. Fei T. Han J. D. Chen Y. G. (2007). MCP-1 mediates TGF-β-induced angiogenesis by stimulating vascular smooth muscle cell migration. *Blood* 109 987–994. 10.1182/blood-2006-07-036400 17032917

[B70] Mariathasan S. Turley S. J. Nickles D. Castiglioni A. Yuen K. Wang Y. (2018). TGFβ attenuates tumour response to PD-L1 blockade by contributing to exclusion of T cells. *Nature* 554 544–548. 10.1038/nature25501 29443960 PMC6028240

[B71] Matthews E. Yang T. Janulis L. Goodwin S. Kundu S. D. Karpus W. J. (2000). Down-regulation of TGF-β1 production restores immunogenicity in prostate cancer cells. *Br. J. Cancer* 83 519–525. 10.1054/bjoc.2000.1257 10945501 PMC2374659

[B72] Moore K. M. Thomas G. J. Duffy S. W. Warwick J. Gabe R. Chou P. (2014). Therapeutic targeting of integrin αvβ6 in breast cancer. *J. Natl. Cancer Inst.* 28:dju169. 10.1093/jnci/dju169 24974129 PMC4151855

[B73] Mori S. Kodaira M. Ito A. Okazaki M. Kawaguchi N. Hamada Y. (2015). Enhanced expression of integrin αvβ3 induced by TGF-β is required for the enhancing effect of fibroblast growth factor 1 (FGF1) in TGF-β-induced epithelial-mesenchymal transition (EMT) in mammary epithelial cells. *PLoS One* 3:e0137486. 10.1371/journal.pone.0137486 26334633 PMC4559424

[B74] Muraoka R. S. Dumont N. Ritter C. A. Dugger T. C. Brantley D. M. Chen J. (2002). Blockade of TGF-β inhibits mammary tumor cell viability, migration, and metastases. *J. Clin. Invest.* 109 1551–1559. 10.1172/jci0215234 12070302 PMC151012

[B75] Nagura M. Matsumura N. Baba T. Murakami R. Kharma B. Hamanishi J. (2015). Invasion of uterine cervical squamous cell carcinoma cells is facilitated by locoregional interaction with cancer-associated fibroblasts via activating transforming growth factor-beta. *Gynecol. Oncol.* 136 104–111. 10.1016/j.ygyno.2014.11.075 25434636

[B76] Nakao A. Afrakhte M. Morén A. Nakayama T. Christian J. L. Heuchel R. (1997). Identification of Smad7, a TGFβ-inducible antagonist of TGF-β signalling. *Nature* 389 631–635. 10.1038/39369 9335507

[B77] Nicolás F. J. Hill C. S. (2003). Attenuation of the TGF-β-Smad signaling pathway in pancreatic tumor cells confers resistance to TGF-β-induced growth arrest. *Oncogene* 22 3698–3711. 10.1038/sj.onc.1206420 12802277

[B78] Nieto M. A. Huang R. Y. Jackson R. A. Thiery J. P. (2016). EMT: 2016. *Cell* 166 21–45. 10.1016/j.cell.2016.06.028 27368099

[B79] Ostrowski M. Carmo N. B. Krumeich S. Fanget I. Raposo G. Savina A. (2010). Rab27a and Rab27b control different steps of the exosome secretion pathway. *Nat. Cell. Biol.* 12(Suppl.1–13), 19–30. 10.1038/ncb2000 19966785

[B80] Poggio M. Hu T. Pai C. C. Chu B. Belair C. D. Chang A. (2019). Suppression of exosomal PD-L1 induces systemic anti-tumor immunity and memory. *Cell* 177 414.e13–427.e13. 10.1016/j.cell.2019.02.016 30951669 PMC6499401

[B81] Rausch M. P. Hahn T. Ramanathapuram L. Bradley-Dunlop D. Mahadevan D. Mercado-Pimentel M. E. (2009). An orally active small molecule TGF-β receptor I antagonist inhibits the growth of metastatic murine breast cancer. *Anticancer Res.* 29 2099–2109. 19528470 PMC2860108

[B82] Ravi R. Noonan K. A. Pham V. Bedi R. Zhavoronkov A. Ozerov I. V. (2018). Bifunctional immune checkpoint-targeted antibody-ligand traps that simultaneously disable TGFβ enhance the efficacy of cancer immunotherapy. *Nat. Commun.* 9 741–754. 10.1038/s41467-017-02696-6 29467463 PMC5821872

[B83] Ren J. Smid M. Iaria J. Salvatori D. C. F. van Dam H. Zhu H. J. (2019). Cancer-associated fibroblast-derived Gremlin 1 promotes breast cancer progression. *Breast Cancer Res.* 21 109–127. 10.1186/s13058-019-1194-0 31533776 PMC6751614

[B84] Roberts A. B. Anzano M. A. Lamb L. C. Smith J. M. Sporn M. B. (1981). New class of transforming growth factors potentiated by epidermal growth factor: isolation from non-neoplastic tissues. *Proc. Natl. Acad. Sci. U.S.A.* 78 5339–5343. 10.1073/pnas.78.9.5339 6975480 PMC348740

[B85] Roberts A. B. Lamb L. C. Newton D. L. Sporn M. B. De Larco J. E. Todaro G. J. (1980). Transforming growth factors: isolation of polypeptides from virally and chemically transformed cells by acid/ethanol extraction. *Proc. Natl. Acad. Sci. U.S.A.* 77 3494–3498. 10.1073/pnas.77.6.3494 6251462 PMC349643

[B86] Rong L. Li R. Li S. Luo R. (2016). Immunosuppression of breast cancer cells mediated by transforming growth factor-β in exosomes from cancer cells. *Oncol. Lett.* 11 500–504. 10.3892/ol.2015.3841 26870240 PMC4727188

[B87] Roth P. Silginer M. Goodman S. L. Hasenbach K. Thies S. Maurer G. (2013). Integrin control of the transforming growth factor-β pathway in glioblastoma. *Brain* 136 564–576. 10.1093/brain/aws351 23378223

[B88] Sachdeva R. Wu M. Johnson K. Kim H. Celebre A. Shahzad U. (2019). BMP signaling mediates glioma stem cell quiescence and confers treatment resistance in glioblastoma. *Sci. Rep.* 9 14569–14582. 10.1038/s41598-019-51270-1 31602000 PMC6787003

[B89] Sadallah S. Amicarella F. Eken C. Iezzi G. Schifferli J. A. (2014). Ectosomes released by platelets induce differentiation of CD4+T cells into T regulatory cells. *Thromb. Haemost.* 112 1219–1229. 10.1160/TH14-03-0281 25209750

[B90] Sadallah S. Schmied L. Eken C. Charoudeh H. N. Amicarella F. Schifferli J. A. (2016). Platelet-derived ectosomes reduce NK cell function. *J. Immunol.* 197 1663–1671. 10.4049/jimmunol.1502658 27448586

[B91] Schneider D. Tarantola M. Janshoff A. (2012). Dynamics of TGF-β induced epithelial-to-mesenchymal transition monitored by electric cell-substrate impedance sensing. *Biochim. Biophys. Acta* 1813 2099–2107. 10.1016/j.bbamcr.2011.07.016 21839117

[B92] Seoane J. Gomis R. R. (2017). TGF-β family signaling in tumor suppression and cancer progression. *Cold Spring Harb. Perspect. Biol.* 9:a022277. 10.1101/cshperspect.a022277 28246180 PMC5710110

[B93] Shi M. Zhu J. Wang R. Chen X. Mi L. Walz T. (2011). Latent TGF-β structure and activation. *Nature* 474 343–349. 10.1038/nature10152 21677751 PMC4717672

[B94] Shi Y. Hata A. Lo R. S. Massagué J. Pavletich N. P. (1997). A structural basis for mutational inactivation of the tumour suppressor Smad4. *Nature* 388 87–93. 10.1038/40431 9214508

[B95] Sorre B. Warmflash A. Brivanlou A. H. Siggia E. D. (2014). Encoding of temporal signals by the TGF-β pathway and implications for embryonic patterning. *Dev. Cell.* 30 334–342. 10.1016/j.devcel.2014.05.022 25065773 PMC4165855

[B96] Sow H. S. Ren J. Camps M. Ossendorp F. Ten Dijke P. (2019). Combined inhibition of TGF-β signaling and the PD-L1 immune checkpoint is differentially effective in tumor models. *Cells* 8:320. 10.3390/cells8040320 30959852 PMC6523576

[B97] Stehelin D. Varmus H. E. Bishop J. M. Vogt P. K. (1976). DNA related to the transforming gene(s) of avian sarcoma viruses is present in normal avian DNA. *Nature* 260 170–173. 10.1038/260170a0 176594

[B98] Stenvers K. L. Tursky M. L. Harder K. W. Kountouri N. Amatayakul-Chantler S. Grail D. (2003). Heart and liver defects and reduced transforming growth factor β2 sensitivity in transforming growth factor β type III receptor-deficient embryos. *Mol. Cell Biol.* 23 4371–4385. 10.1128/mcb.23.12.4371-4385.2003 12773577 PMC156130

[B99] Stockis J. Liénart S. Colau D. Collignon A. Nishimura S. L. Sheppard D. (2017). Blocking immunosuppression by human Tregs in vivo with antibodies targeting integrin αVβ8. *Proc. Natl. Acad. Sci. U.S.A.* 114 E10161–E10168. 10.1073/pnas.1710680114 29109269 PMC5703296

[B100] Suzuki E. Kim S. Cheung H. K. Corbley M. J. Zhang X. Sun L. (2007). A novel small-molecule inhibitor of transforming growth factor beta type I receptor kinase (SM16) inhibits murine mesothelioma tumor growth in vivo and prevents tumor recurrence after surgical resection. *Cancer Res.* 67 2351–2359. 10.1158/0008-5472.can-06-2389 17332368

[B101] Taipale J. Miyazono K. Heldin C. H. Keski-Oja J. (1994). Latent transforming growth factor-beta 1 associates to fibroblast extracellular matrix via latent TGF-beta binding protein. *J. Cell Biol.* 124 171–181. 10.1083/jcb.124.1.171 8294500 PMC2119892

[B102] Takasaka N. Seed R. I. Cormier A. Bondesson A. J. Lou J. Elattma A. (2018). Integrin αvβ8-expressing tumor cells evade host immunity by regulating TGF-β activation in immune cells. *JCI Insight* 3 122591–122608. 10.1172/jci.insight.122591 30333313 PMC6237456

[B103] Tang F. Wang H. Chen E. Bian E. Xu Y. Ji X. (2019). LncRNA-ATB promotes TGF-β-induced glioma cells invasion through NF-κB and P38/MAPK pathway. *J. Cell Physiol.* 234 23302–23314. 10.1002/jcp.28898 31140621

[B104] Tauriello D. Palomo-Ponce S. Stork D. Berenguer-Llergo A. Badia-Ramentol J. Iglesias M. (2018). TGFβ drives immune evasion in genetically reconstituted colon cancer metastasis. *Nature* 554 538–543. 10.1038/nature25492 29443964

[B105] Thomas D. A. Massagué J. (2005). TGF-β directly targets cytotoxic T cell functions during tumor evasion of immune surveillance. *Cancer Cell* 8 369–380. 10.1016/j.ccr.2005.10.012 16286245

[B106] Todaro G. J. De Larco J. E. Cohen S. (1976). Transformation by murine and feline sarcoma viruses specifically blocks binding of epidermal growth factor to cells. *Nature* 264 26–31. 10.1038/264026a0 187944

[B107] van Caam A. Aarts J. van Ee T. Vitters E. Koenders M. van de Loo F. (2020). TGFβ-mediated expression of TGFβ-activating integrins in SSc monocytes: disturbed activation of latent TGFβ? *Arthritis Res. Ther*. 22 42–50. 10.1186/s13075-020-2130-5 32143707 PMC7059334

[B108] Vollaire J. Machuca-Gayet I. Lavaud J. Bellanger A. Bouazza L. El Moghrabi S. (2019). The bone morphogenetic protein signaling inhibitor LDN-193189 enhances metastasis development in mice. *Front. Pharmacol.* 10:667. 10.3389/fphar.2019.00667 31275146 PMC6593094

[B109] Walton K. L. Makanji Y. Chen J. Wilce M. C. Chan K. L. Robertson D. M. (2010). Two distinct regions of latency-associated peptide coordinate stability of the latent transforming growth factor-β1 complex. *J. Biol. Chem.* 285 17029–17037. 10.1074/jbc.M110.110288 20308061 PMC2878044

[B110] Wang J. Tucker-Kellogg L. Ng I. C. Jia R. Thiagarajan P. S. White J. K. (2014). The self-limiting dynamics of TGF-β signaling in silico and in vitro, with negative feedback through PPM1A upregulation. *PLoS Comput. Biol.* 10:e1003573. 10.1371/journal.pcbi.1003573 24901250 PMC4105941

[B111] Warmflash A. Zhang Q. Sorre B. Vonica A. Siggia E. D. Brivanlou A. H. (2012). Dynamics of TGF-β signaling reveal adaptive and pulsatile behaviors reflected in the nuclear localization of transcription factor Smad4. *Proc. Natl. Acad. Sci. U.S.A.* 109 E1947–E1956. 10.1073/pnas.1207607109 22689943 PMC3396545

[B112] Webber J. Steadman R. Mason M. D. Tabi Z. Clayton A. (2010). Cancer exosomes trigger fibroblast to myofibroblast differentiation. *Cancer Res.* 70 9621–9630. 10.1158/0008-5472.CAN-10-1722 21098712

[B113] Wegner K. Bachmann A. Schad J. U. Lucarelli P. Sahle S. Nickel P. (2012). Dynamics and feedback loops in the transforming growth factor β signaling pathway. *Biophys. Chem.* 162 22–34. 10.1016/j.bpc.2011.12.003 22284904

[B114] Xia Y. Zhang Q. Zhen Q. Zhao Y. Liu N. Li T. (2017). Negative regulation of tumor-infiltrating NK cell in clear cell renal cell carcinoma patients through the exosomal pathway. *Oncotarget* 8 37783–37795. 10.18632/oncotarget.16354 28384121 PMC5514949

[B115] Yang J. Lu Y. Lin Y. Y. Zheng Z. Y. Fang J. H. He S. (2016). Vascular mimicry formation is promoted by paracrine TGF-β and SDF1 of cancer-associated fibroblasts and inhibited by miR-101 in hepatocellular carcinoma. *Cancer Lett.* 383 18–27. 10.1016/j.canlet.2016.09.012 27693460

[B116] Yang L. Huang J. Ren X. Gorska A. E. Chytil A. Aakre M. (2008). Abrogation of TGF β signaling in mammary carcinomas recruits Gr-1+CD11b+ myeloid cells that promote metastasis. *Cancer Cell* 13 23–35. 10.1016/j.ccr.2007.12.004 18167337 PMC2245859

[B117] Yang L. Pang Y. Moses H. L. (2010). TGF-β and immune cells: an important regulatory axis in the tumor microenvironment and progression. *Trends Immunol.* 31 220–227. 10.1016/j.it.2010.04.002 20538542 PMC2891151

[B118] Yang Y. A. Dukhanina O. Tang B. Mamura M. Letterio J. J. MacGregor J. (2002). Lifetime exposure to a soluble TGF-β antagonist protects mice against metastasis without adverse side effects. *J. Clin. Invest.* 109 1607–1615. 10.1172/jci200215333 12070308 PMC151015

[B119] Yen E. Y. Miaw S. C. Yu J. S. Lai I. R. (2017). Exosomal TGF-β1 is correlated with lymphatic metastasis of gastric cancers. *Am. J. Cancer Res.* 7 2199–2208. 29218244 PMC5714749

[B120] Yu Y. Xiao C. H. Tan L. D. Wang Q. S. Li X. Q. Feng Y. M. (2014). Cancer-associated fibroblasts induce epithelial-mesenchymal transition of breast cancer cells through paracrine TGF-β signalling. *Br. J. Cancer* 110 724–732. 10.1038/bjc.2013.768 24335925 PMC3915130

[B121] Zhang H. T. Fei Q. Y. Chen F. Qi Q. Y. Zou W. Wang J. C. (2003). Mutational analysis of the transforming growth factor β receptor type I gene in primary non-small cell lung cancer. *Lung Cancer* 40 281–287. 10.1016/s0169-5002(03)00121-1 12781426

[B122] Zhang M. Kleber S. Röhrich M. Timke C. Han N. Tuettenberg J. (2011). Blockade of TGF-β signaling by the TGFβR-I kinase inhibitor LY2109761 enhances radiation response and prolongs survival in glioblastoma. *Cancer Res.* 71 7155–7167. 10.1158/0008-5472.CAN-11-1212 22006998

[B123] Zhang S. Fei T. Zhang L. Zhang R. Chen F. Ning Y. (2007). Smad7 antagonizes transforming growth factor β signaling in the nucleus by interfering with functional Smad-DNA complex formation. *Mol. Cell Biol.* 27 4488–4499. 10.1128/mcb.01636-06 17438144 PMC1900056

[B124] Zhong Z. Carroll K. D. Policarpio D. Osborn C. Gregory M. Bassi R. (2010). Anti-transforming growth factor beta receptor II antibody has therapeutic efficacy against primary tumor growth and metastasis through multieffects on cancer, stroma, and immune cells. *Clin. Cancer Res.* 16 1191–11205. 10.1158/1078-0432.CCR-09-1634 20145179

[B125] Zhou F. Drabsch Y. Dekker T. J. de Vinuesa A. G. Li Y. Hawinkels L. J. (2014). Nuclear receptor NR4A1 promotes breast cancer invasion and metastasis by activating TGF-β signalling. *Nat. Commun.* 5 3388–3400. 24584437 10.1038/ncomms4388

[B126] Zhu H. Gu X. Xia L. Zhou Y. Bouamar H. Yang J. (2018). A novel TGFβ trap blocks chemotherapeutics-induced TGFβ1 signaling and enhances their anticancer activity in gynecologic cancers. *Clin. Cancer Res.* 24 2780–2793. 10.1158/1078-0432.CCR-17-3112 29549162 PMC6004245

[B127] Zhu H. J. Sizeland A. M. (1999). A pivotal role for the transmembrane domain in transforming growth factor-β receptor activation. *J. Biol. Chem.* 274 11773–11781. 10.1074/jbc.274.17.11773 10206994

[B128] Zi Z. Feng Z. Chapnick D. A. Dahl M. Deng D. Klipp E. (2011). Quantitative analysis of transient and sustained transforming growth factor-β signaling dynamics. *Mol. Syst. Biol.* 7 492–503. 10.1038/msb.2011.22 21613981 PMC3130555

